# Instantaneous Clearing of Biofilm (iCBiofilm): an optical approach to revisit bacterial and fungal biofilm imaging

**DOI:** 10.1038/s42003-022-04396-4

**Published:** 2023-01-23

**Authors:** Shinya Sugimoto, Yuki Kinjo

**Affiliations:** 1 Department of Bacteriology, The Jikei University School of Medicine, 3-25-8 Nishi-Shimbashi, Minato-ku, Tokyo 105-8461 Japan; 2 Jikei Center for Biofilm Science and Technology, The Jikei University School of Medicine, 3-25-8 Nishi-Shimbashi, Minato-ku, Tokyo 105-8461 Japan

**Keywords:** Biofilms, Optical imaging, Microbiology techniques, Imaging the immune system

## Abstract

Whole-biofilm imaging at single-cell resolution is necessary for system-level analysis of cellular heterogeneity, identification of key matrix component functions and response to immune cells and antimicrobials. To this end, we developed a whole-biofilm clearing and imaging method, termed instantaneous clearing of biofilm (iCBiofilm). iCBiofilm is a simple, rapid, and efficient method involving the immersion of biofilm samples in a refractive index-matching medium, enabling instant whole-biofilm imaging with confocal laser scanning microscopy. We also developed non-fixing iCBiofilm, enabling live and dynamic imaging of biofilm development and actions of antimicrobials. iCBiofilm is applicable for multicolor imaging of fluorescent proteins, immunostained matrix components, and fluorescence labeled cells in biofilms with a thickness of several hundred micrometers. iCBiofilm is scalable from bacterial to fungal biofilms and can be used to observe biofilm-neutrophil interactions. iCBiofilm therefore represents an important advance for examining the dynamics and functions of biofilms and revisiting bacterial and fungal biofilm formation.

## Abstract (web summary)

A new clearing and imaging technique termed iCBiofilm is developed for imaging whole bacterial and fungal biofilms using confocal laser scanning microscopy, which enables examining the dynamics and functions of biofilms.

## Introduction

Bacterial biofilms are highly organized bacterial communities formed on abiotic or biotic surfaces and air-liquid interfaces. Bacterial biofilms on the surfaces of medical implants and body tissues can be resistant to antimicrobial agents and host defense systems, including phagocytes, complements, and antimicrobial peptides. This phenomenon causes various chronic human infectious diseases, such as catheter-related bloodstream infections, urinary tract infections, and endocarditis^1–3^. The formation of biofilm in clinical settings, therefore, results in increased morbidity and mortality, imposing a significant financial burden on the healthcare system. In addition, biofilms can cause logistical problems in technical operations, such as those encountered in the maintenance of drinking water distribution systems^4^. However, biofilms also possess beneficial features for human industry, such as those exploited by fermented food production, bioconversion processes for organic compounds, and wastewater treatment^5^. The development of innovative strategies for the control and analysis of biofilm formation is therefore significant for various fields.

Bacterial cells in biofilms are believed to be encased in a typically self-produced matrix comprising extracellular polymeric substances (EPS) such as proteins, polysaccharides, and/or extracellular DNA^6^. EPS plays diverse roles in the formation and maintenance of biofilm structures via the stimulation of microbe-microbe cohesion and microbe-surface adhesion^6^. However, the three-dimensional distributions of biofilm matrix components and heterogeneous properties of embedded bacterial cells are not fully understood. Moreover, heterogeneity or cellular differentiation in biofilms is a commonly accepted concept, but direct evidence on the microscopic scale has been difficult to obtain in the case of thick biofilms.

The development of strategies that enable rapid and efficient biofilm visualization is, therefore, necessary to better understand their structures and functions, including interactions with other microbes and the host and responses to antimicrobials. Whereas optical microscopy (OM) such as confocal laser scanning microscopy (CLSM) and light-sheet microscopy combined with fluorescence probes enabled researchers to study the three-dimensional biofilm architecture and to make a substantial contribution to the current understanding of biofilms^7–10^, imaging of thick biofilms at a single-cell level remains challenging, as the penetration of light into deeper regions is limited when using conventional OM techniques. In addition, standard CLSM using a water immersion lens enabled live single-cell resolution imaging of a *Vibrio cholera* biofilm^11,12^. This method may be applicable to the imaging of other bacterial biofilms.

Tissue clearing is a process that removes biomacromolecules (mainly lipids) causing light refraction and scattering, thereby adjusting the refractive index (RI) of the tissues in a surrounding solvent. In the field of neuroscience, three-dimensional imaging based on tissue clearing is the most practical strategy for visualizing single cells within opaque biological samples, including whole organs and animal bodies^13^. The basic principle of human tissue clearing using organic solvents was introduced almost 100 years ago^14^. Various tissue-clearing methods have since been developed, including (i) organic solvent-based methods, such as BABB^15^, 3DISCO^16^, and iDISCO;^17^ (ii) hydrophilic chemical-based methods such as SeeDB^18^, CUBIC^19^, and Sca*l*e;^20^ and (iii) hydrogel-base methods, including CLARITY^21^, PACT^22^, and SWITCH^23^. Although various tissue-clearing methods have become available over the last 10 years, as far as we known, they have not yet been applied to the analysis of biofilm structure, function, and physiology. Furthermore, tissue clearing-based live-cell imaging remains a challenge because typical clearing methods require sample fixation and time-consuming clearing and decoloring procedures, which take several days or more than a week to conduct.

In this study, we developed an ‘instantaneous clearing of biofilm (iCBiofilm)’ method using RI-matching media to examine thick biofilms formed by gram-positive and negative bacteria, as well as the eukaryotic *Candida albicans* at a single-cell resolution. iCBiofilm enables dynamic and live imaging of biofilm development and antimicrobial agent activity. Using this method, we demonstrate heterogeneous distributions of biofilm matrix components in *S. aureus* biofilms and propose an underlying mechanism for how the spatial distributions of biofilm matrix proteins are determined. iCBiofilm is also useful for imaging biofilm-neutrophil interactions.

## Results and discussion

### iCBiofilm as a non-invasive approach to image biofilm three-dimensional structure

Of the various tissue-clearing reagents recently developed, we deemed SeeDB2 the most suitable for visualizing thick biofilms at a single-cell resolution because it is a simple soaking-based and morphology-preserving product involving high concentrations of iohexol^24^. Therefore, as a preliminary experiment, we selected SeeDB2 to image a thick biofilm formed by the model microorganism *Staphylococcus aureus*. Throughout this study, we mainly used *S. aureus* MR23, a clinically isolated, methicillin-resistant strain that forms a thick biofilm in a brain heart infusion medium supplemented with 1% glucose (BHIG)^9,25,26^. We used inverted microscopes to visualize the three-dimensional structures of biofilms formed on the surface of 35-mm glass-bottomed dishes.

**Fig. 1:**
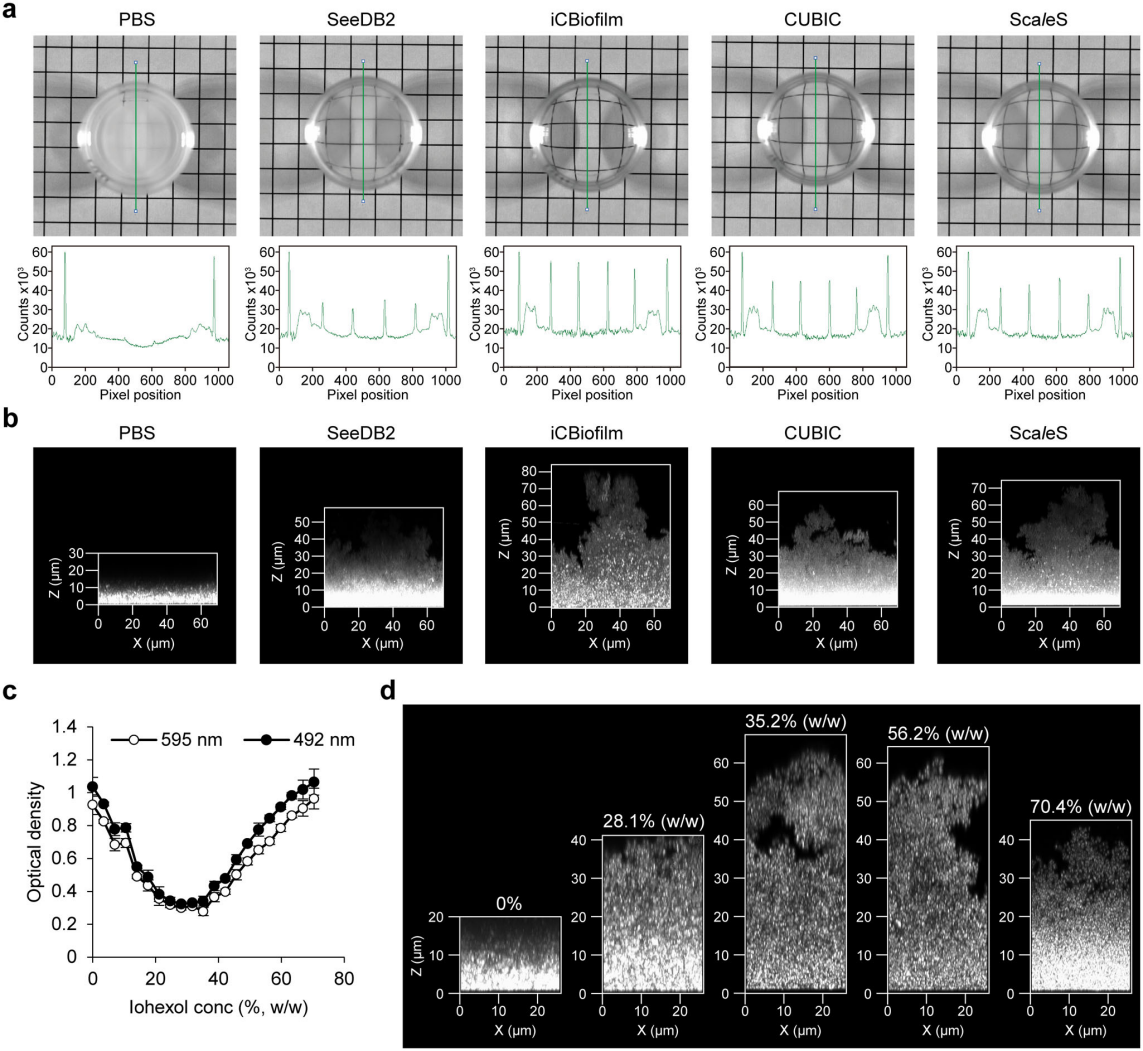
Development of the iCBiofilm method. **a** Photographs of MR23 biofilms on a grid pattern-printed paper as a background was obtained using ImageQuant for transmission. Plot profiles for the biofilms treated with the indicated tissue-clearing reagents are also shown in the lower panels. The biofilm treated with phosphate-buffered saline (PBS) was used as the control. The green line in the upper image indicates the corresponding location of the plot profile in the lower panel, respectively. **b** Typical side views of the MR23 biofilms stained with thioflavin T (ThT). An LSM880 confocal laser scanning microscope with a ×40 oil immersion objective lens and an Airyscan super-resolution unit (Carl Zeiss, Oberkochen, Germany) was used to acquire z-stacks of the stained biofilms every 0.22 μm. **c** Optical density vs iohexol concentration for MR23 biofilms. Means and standard deviations from three measurements are shown. **d** Typical side views of fixed MR23 biofilms stained with FM1-43 and soaked in the indicated iohexol solutions. LSM880 confocal laser scanning microscope with ×63 objective lens and Airyscan super-resolution unit was used to acquire z-stacks of the stained biofilms every 0.18 μm. X and Z represent width and thickness, respectively.

The mature biofilm of MR23 was opaque in phosphate-buffered saline (PBS) but was transparent after treatment with SeeDB2 (Fig. 1a). Inverted CLSM revealed that, when soaked in PBS, only cells located close to the bottom of the biofilm, within 10 μm of the glass surface, were visible due to high light scattering and spherical aberrations (Fig. 1b). It should be noted that a smooth transition in the z-direction was observed, indicating the presence of bacterial cells, but not the absence of cells, above the visible region. In contrast, when biofilms were treated with SeeDB2 and stained with thioflavin T (ThT), which stains bacterial RNA^27^, the upper region became visible to some extent, with a visible thickness of approximately 40 μm (Fig. 1b). However, we noticed that the clearing capacity of SeeDB2 was not enough, and that repeated reagent replacement removed some cells from the biofilm. In addition, saponin (the detergent included in SeeDB2) was suspected to destroy the biofilm structure, since detergents in general can affect the biofilm structure. Therefore, we aimed to develop an optical clearing method optimized for biofilms using a detergent-free reagent.

During the procedure of biofilm clearing using SeeDB2, we noticed that the transparency of the *S. aureus* biofilm changed from opaque to transparent in the clearing step using Clearing Solutions 1 and 2, then reverted to slightly opaque in the steps using Clearing Solutions 3 and 4. This observation suggested that the RIs of clearing solution 3 (RI = 1.46) and 4 (RI = 1.52) were higher than those of the *S. aureus* biofilm. Consistent with this, the RI of many bacteria is approximately 1.38–1.42^28^. Therefore, we optimized the concentration of iohexol for the *S. aureus* biofilm by reducing its concentration. To this end, we developed a high-throughput screening method using 96-well plastic plates and a microphotometer to measure the optical densities of non-fixed *S. aureus* biofilms soaked in various concentrations of iohexol. The optical density of the biofilm changed in an iohexol concentration-dependent manner (Fig. 1c). In addition, fixation with 1% glutaraldehyde (GA) affected the chemical characteristics of the biofilm, increasing the iohexol concentration required for optimal transparency (Supplementary Fig. 1a). Importantly, high concentrations of iohexol dispersed the biofilm, resulting in decreased biomass, whereas fixation with GA stabilized it (Supplementary Fig. 1b). Therefore, biofilms in subsequent experiments were fixed before soaking in iohexol. Following this procedure, the lowest optical density of the GA-fixed biofilm was achieved with 30–50% (w/w) iohexol (Supplementary Fig. 1a). CLSM imaging of an *S. aureus* biofilm stained with the membrane dye FM1-43 also revealed that biofilms with >60 μm thickness were clearly visualized in 35.2% (w/w) iohexol (Fig. 1d). Similarly, a ThT-stained biofilm was clearly observed at all thicknesses with single-cell resolution (Supplementary Movie 1). Most notably, the opaque biofilm became transparent immediately after adding 35.2% (w/w) iohexol alone (Supplementary Movie 2), indicating that the RI-matching medium was sufficient to lend transparency to the *S. aureus* biofilm without removing some biomolecules. This simple and efficient method to visualize thick biofilms using a detergent-free, RI-matching reagent alone was termed iCBiofilm. Of note, the RI-matching reagent is not limited to iohexol (Supplementary Fig. 2 and 3). Diatrizoate, iodixanol, iopromide, iopamidol, and ioversol showed high clearing capacity (Supplementary Fig. 2a). These reagents improved imaging of the thick biofilm compared with PBS (Supplementary Fig. 2b). Previously, a water immersion lens was used to analyze dynamics of a live *V. cholera* biofilm^11,12^, but we could not observe any differences in the visible thickness between oil and water immersion lenses, at least in the case of the PBS-immersed *S. aureus* biofilm (Supplementary Fig. 2b). Therefore, we think that matching of RI between biofilms and the surrounding solution is more important for whole-biofilm imaging rather than the choice of an objective lens. As mentioned below, the RI-matching reagent can be changed for experimental purposes.

Next, we compared iCBiofilm with other available tissue-clearing methods including CUBIC^19^, Sca*l*eS^29^, and SeeDB2^24^ for clearing and imaging of the MR23 biofilm. Biofilm clearing capacity of iCBiofilm was superior to those of the others (Fig. 1a). iCBiofilm greatly improved imaging of the MR23 biofilm with a thickness of ~80 μm, while the other methods were insufficient for whole-biofilm imaging (Fig. 1b). Although CUBIC, Sca*l*eS, and SeeDB2 required several days or weeks for clearing of biofilms and tissues, iCBiofilm took only several seconds for biofilm clearing (Supplementary Movie 2). iCBiofilm preserved structure of the fixed biofilm, while SeeDB2 damaged it probably due to repeated replacement of viscous clearing reagents supplemented with detergents. In addition, handling of iCBiofilm was very easy, while CUBIC, Sca*l*eS, and SeeD2 were easy but contained viscous reagents, which made handling slightly difficult. Collectively, iCBiofilm was the most rapid, simple, effective, and non-invasive method for whole-biofilm imaging (Table 1).

**Table 1:** Comparison of iCBiofilm properties with other tissue-clearing methods.

Method	Sample	Agents		Refractive index	Clearing capacity	Time	Handling	Biofilm structure	Live-cell imaging	Reference
		Main	Detergent							
iCBiofilm	Biofilm	Diatrizoate 47.2% (w/v)	–	1.44^a^	Strong^d^	Several seconds	Easy but causing decolorization	Preserved (only for fixed biofilms)	N.T.	This study
	Biofilm	Iohexol 35.2–56.2% (w/w)	–	1.39–1.43^a^	Strong^d^	Rapid	Very easy	Preserved (only for fixed biofilms)	Difficult	This study
	Biofilm	Iodixanol 15–45% (w/v)	–	1.36–1.40^a^	Strong^d^	Several seconds	Very easy	Preserved	Possible	This study
	Biofilm	Ioversol 37.1–55.6%	–	1.39–1.43^a^	Strong^d^	Several seconds	Very easy	Preserved (only for fixed biofilms)	N.T.	This study
	Biofilm	Iopamidol 56.7% (w/v)	–	1.43^a^	Strong^d^	Several seconds	Very easy	Preserved (only for fixed biofilms)	N.T.	This study
	Biofilm	Iopromide 57.7% (w/v)	–	1.42^a^	Strong^d^	Several seconds	Very easy	Preserved (only for fixed biofilms)	N.T.	This study
CUBIC	Mouse Marmoset	Urea Amino alcohol	Triton X-100	1.49^b^	Strong^e^	1–2 Weeks^e^	Easy but viscous for Sca*l*eCUBIC-2 Solution	N.A.	N.A.	19 FUJIFILM Wako
	Biofilm				Medium^d^	4 days		Preserved (only for fixed biofilms)	N.T.	This study
Sca*l*eS	Mouse	Urea Sorbitol	Triton X-100	1.49^b^	Strong^e^	Several days^e^	Easy but viscous for SCALEVIEW-SMt	N.A.	N.A.	20 FUJIFILM Wako
	Biofilm				Medium^d^	2 days		Preserved (only for fixed biofilms)	N.T.	This study
SeeDB2	Mouse Drosophila Culture cells	Iohexol 56.2–70.4% (w/w)	Saponin	1.46–1.52^c^	Strong^c^	Several days^c^	Easy but viscous for SeeDB2S	N.A.	N.A.	24 FUJIFILM Wako
	Biofilm				Weak^d^	4–5 days		Damaged	N.T.	This study

^a^Refractive indices of the solutions were measured using a refractometer (DR-M2, ATAGO) at 22–23 °C.

^b^Referred to the manufacturers’ instructions.

^c^Referred to Ke et al. 2016^18^.

^d^Clearing capability was categorized into strong (>75%), medium (50–75%), and weak (<50%) according to the optical transmittance measurement of MR23 biofilms (Fig. 1e and Supplementary Fig. 2a).

^e^Referred to Hama et al. 2015^20^.

N.A. not applicable, N.T. not tested.

### iCBiofilm enables live and dynamic imaging of biofilm development

Bacterial biofilm formation is believed to begin with initial contact of an individual bacterial cell with a surface, followed by proliferation and formation of a micro-colony, production of extracellular matrices that embed bacterial cells, and establishment of a complex, three-dimensional structure to form a mature biofilm^5^. To confirm whether this widely-accepted theory could be observed using our method, we performed live-cell imaging for biofilm development using iCBiofilm. First, we examined the cytotoxicity of iohexol against *S. aureus*. Iohexol reduced the turbidity of the liquid culture but did not affect the viability of *S. aureus* (Supplementary Fig. 4a, b).

**Fig. 2:**
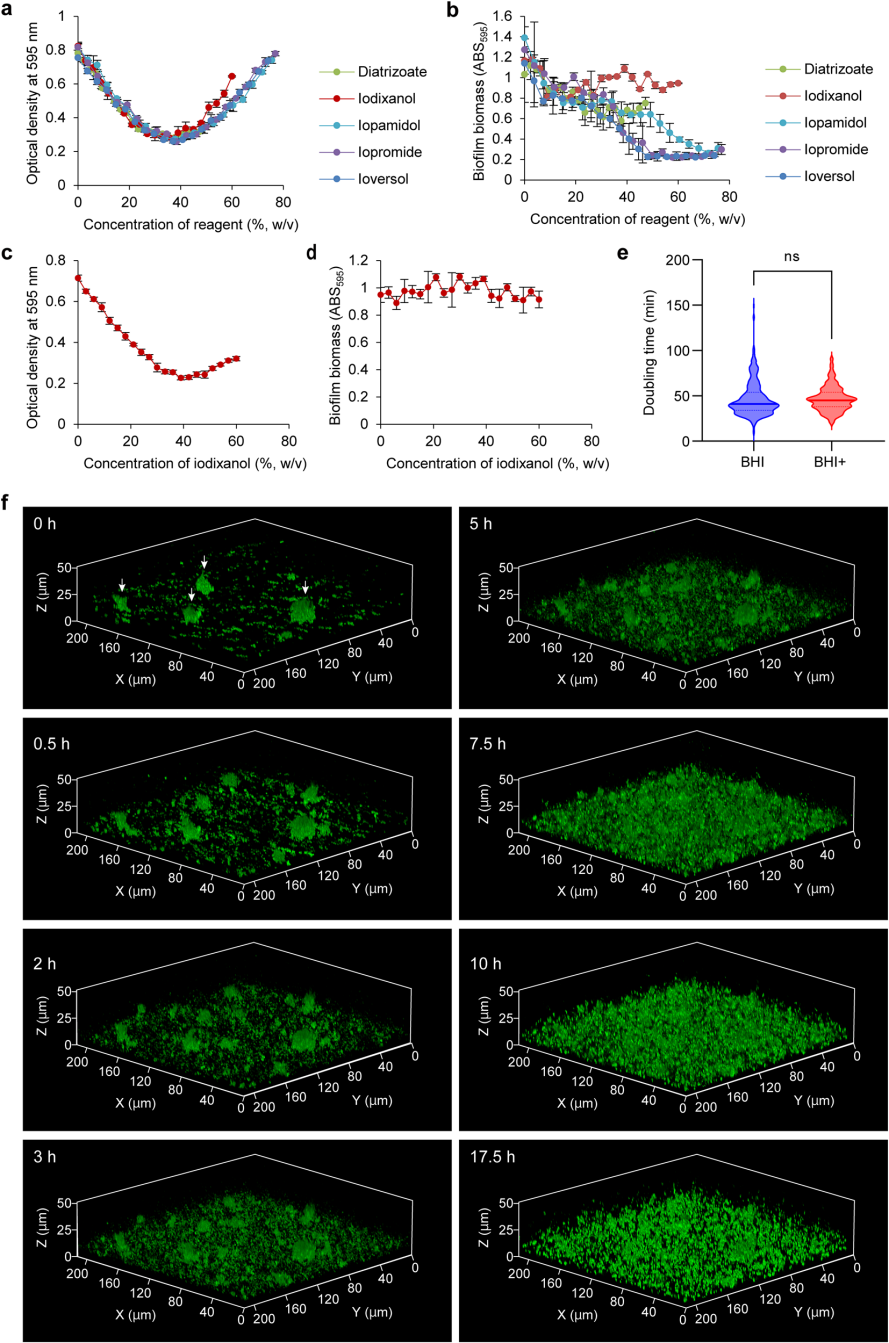
Live-cell imaging with iCBiofilm. **a** Optical density vs concentration of the indicated refractive index-matching media for non-fixed *S. aureus* MR4 biofilm. **b** Effects of the clearing reagents on stability of non-fixed MR4 biofilm. **c** Optical density vs iodixanol concentration for non-fixed *S. epidermidis* SE21 biofilm. **d** Effects of iodixanol on stability of non-fixed SE21 biofilm. **e** Violin plots of the doubling times of SE21 cells measured by optical microscopy at the single-cell level. Cells were grown on BHI agar plates in the absence (BHI, *n* = 403) and presence of 30.0% (w/v) iodixanol (BHI+, *n* = 283) under the phase-contrast microscope. ns, not significant (unpaired two-tailed Student *t* test). **f** Live-cell clearing imaging for biofilm formation of *S. epidermidis* SE21 in BHIG containing 30.0% (w/v) iodixanol and 25 μM ThT in a glass-bottomed dish. 3D images at the indicated time points are shown. Arrows indicate cell clusters formed at the initial phase of biofilm formation. Means and standard deviations from four measurements are shown in **a**–**d**.

Next, we examined effects of iohexol on the biofilm structure of *S. aureus* MR23. When cultured in BHIG, a robust biofilm of MR23 formed, tightly attached to the glass surface (Supplementary Fig. 5). However, when cultured in BHIG supplemented with 28.1% (w/w) iohexol, a floating, fragile biofilm was observed (Supplementary Fig. 5). This indicated that 28.1% (w/w) iohexol interfered with the interaction of the biofilm with the glass surface, thus severely affecting adhesion and structure. Therefore, we looked for RI-matching media optimal for live-cell imaging of fragile biofilms using *S. aureus* MR4 as an indicator strain with a biofilm more fragile than that of *S. aureus* MR23. All the tested compounds achieved biofilm clearing to a similar extent (Fig. 2a), but only iodixanol did not show a remarkably destructive effect on the pre-formed MR4 biofilm (Fig. 2b), suggesting that iodixanol can be used for various types of biofilms. Importantly, this compound did not affect the viability, growth rate, and cell shape of *S. aureus* (Supplementary Fig. 4b–d). In addition, iodixanol was able to clear the biofilm of *S. epidermidis* SE21, a clinical isolate that forms a polysaccharide-dependent biofilm (Fig. 2c) and did not cause dispersion in the pre-formed biofilm (Fig. 2d). Growth rate of *S. epidermidis* SE21 was also not affected by iodixanol (Fig. 2e). Therefore, we selected iodixanol for live-cell imaging of biofilm development.

To perform live-cell imaging stably over time using the ZEISS LSM880 confocal microscope, biofilms were incubated for 2–4 h in an incubation chamber on a confocal microspore equipped with an autofocus module. First, ThT was used to stain *S. epidermidis* SE21 cells because it does not inhibit growth at an optimized concentration^27^. ThT-stained individual cells and microcolonies were readily observed at the onset of imaging (Fig. 2f and Supplementary Movie 3). Unexpectedly, proliferation of cells in addition to microcolonies was observed over time, rather than the expansion of microcolonies (Fig. 2f and Supplementary Movie 3). This was not a side effect of ThT, as confocal reflection microscopy of a non-stained *S. epidermidis* biofilm showed a similar trend (Supplementary Movie 4). To expand the applicability of iCBiofilm-based live-cell imaging, we also used MitoTracker Deep Red and the THUNDER Imaging System developed by Leica Microsystems to perform quick 3D live-cell imaging. MitoTracker fluorescent dyes are often used to stain mitochondria in eukaryotic cells depending on their membrane potential and are able to stain living bacteria without affecting the growth rate^30^. Because of the rapid imaging speed, the THUNDER imaging system allowed us to view the accumulation of planktonic cells on the glass surface and on the pre-existing biofilm (Supplementary Movie 5). As in the case of LSM880 imaging, the proliferation of cells adjacent to microcolonies, rather than expansion of pre-existing microcolonies, was predominantly observed over time (Supplementary Movie 5).

Live-cell imaging using iCBiofilm was also performed to analyze biofilm formation by *S. aureus* MR23. Interestingly, expansion of microcolonies accompanied by the accumulation of planktonic and aggregated cells above the biofilm was observed (Supplementary Fig. 6 and Supplementary Movie 6), which was inconsistent with the biofilm formation process of *S. epidermidis* SE21 described above (Fig. 2f and Supplementary Movies 3–5). A slight decay of fluorescence was observed, likely due to a reduction in membrane potential as metabolic activity within the biofilm decreased^31^. In addition, biofilm formation by *S. aureus* MR4 was visualized by iCBiofilm-based live cell imaging (Supplementary Movie 7), and the process was distinct from that of SE21 or MR23 (Supplementary Movies 5 and 6). Specifically, highly dynamic and mobile cells were observed during biofilm formation by MR4 (Supplementary Movie 7). These results indicate that the process of biofilm development differs among bacterial species and strains. Live-cell imaging using iCBiofilm is therefore useful to analyze biofilm formation across various biofilm types.

### iCBiofilm enables the imaging of the impact of antimicrobials on bacterial biofilm

Given our findings indicating that iCBiofilm can achieve live-cell imaging, we aimed to observe the effects of antimicrobials on a living biofilm. Syto 9 and propidium iodide (PI) were used to distinguish between live and dead (membrane-damaged) cells. Both are nucleic acid dyes, but Syto 9 permeabilizes both live and dead cells, whereas PI only permeabilizes membrane-damaged (dead) cells^32^. This method allowed the stable visualization of live and dead cells within the biofilm for at least 1 h (Supplementary Fig. 7 and Supplementary Movie 8). First, we treated the biofilm with vancomycin, which is effective against planktonic cells of *S. aureus* but not against its biofilm^32^. As expected, vancomycin was not effective in killing *S. aureus* cells within the biofilm (Supplementary Fig. 7 and Supplementary Movie 9). Next, nisin A isolated from *Lactococcus lactis* was used because it is considered effective in killing pre-formed biofilms of various *S. aureus* strains^32^. After adding nisin A, the proportion of dead cells increased rapidly, but not all cells were killed (Supplementary Fig. 7 and Supplementary Movie 10). This was consistent with previous CFU count data, which showed that only 90% of cells in the *S. aureus* MR23 biofilm were killed by nisin A^32^. Interestingly, cells in the convex regions of the biofilm showed tolerance against nisin A compared with those in the concave regions (Supplementary Fig. 7 and Supplementary Movie 10), possibly because of the heterogeneous distribution of susceptible and tolerant cells and the existence of matrix components interfering with the action of nisin A in the convex regions.

Collectively, these results indicate that iCBiofilm is applicable for the imaging of the antimicrobial effects on biofilms and might be useful for establishing medical solutions for biofilm eradication.

### iCBiofilm improves the visualization of major *S. aureus* biofilm matrix proteins

**Fig. 3:**
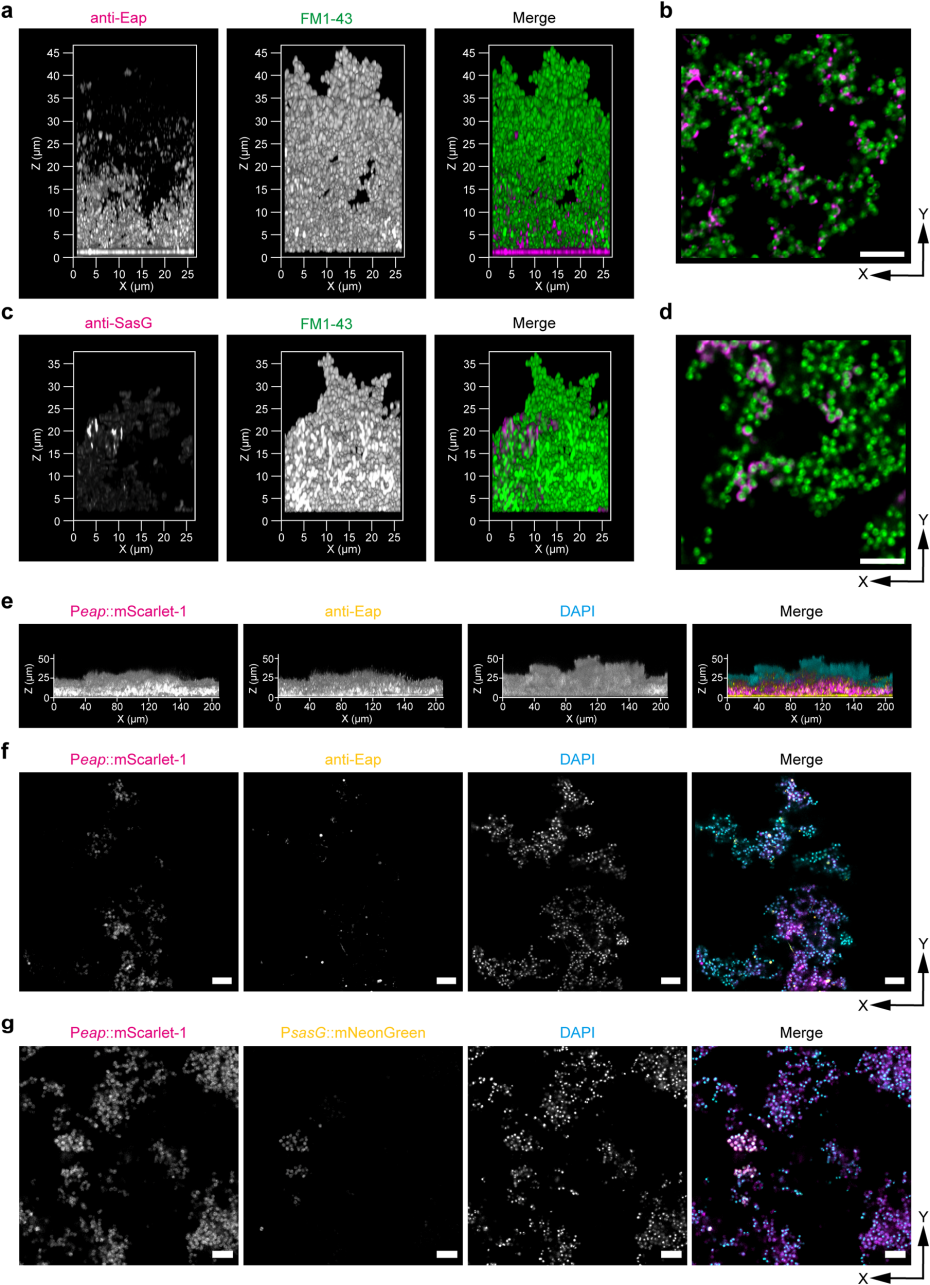
Localization of secreted Eap and cell wall-anchored SasG proteins in the *S. aureus* biofilms, observed using iCBiofilm. The typical side views (**a**, **c**) and the X-Y sections for the selected z-stack positions (**b**, **d**) of MR23 Δ*spa* Δ*sbi* biofilms stained with antibodies and FM1-43. Fluorescent signals from Eap/SasG and cells (membrane) in the merged view are shown in magenta and green, respectively. **e** Side views of the biofilm formed by MR23 Δ*spa* Δ*sbi* P_*eap*_::mScarlet-1. **f** X-Y sections for the selected z-stack showing distribution of Eap and Eap-expressing cells in the biofilm. Fluorescent signals from mScarlet-I (Eap-expressing cells), anti-Eap/Alexa 488-conjugated anti-rabbit IgG, and DNA (total bacterial cells) are shown in magenta, yellow, and cyan, respectively, in **e**, **f**. **g** The X-Y sections for the selected z-stack show distribution of cells expressing Eap and/or SasG in the 24 h biofilm of MR23 Δ*spa* Δ*sbi* P_*eap*_::mScarlet-1 P_*sasG*_::mNeonGreen. Fluorescent signals from mScarlet-I (Eap-expressing cells), mNeonGreen (SasG-expressing cells), and DNA (total bacterial cells) are shown in magenta, yellow, and cyan, respectively. Scale bars are 5 μm. X and Z represent width and thickness, respectively. All images were taken after soaking the biofilms in 35.2% (w/w) iohexol solution. An LSM880 microscope with ×20, ×63, and ×100 objective lenses and an Airyscan super-resolution unit were used to acquire z-stacks of the stained biofilms.

To test whether iCBiofilm could be applied to the visualization of *S. aureus* matrix components, we first attempted to visualize secreted and cell wall-anchored proteins such as extracellular adherence protein (Eap) and *Staphylococcus aureus* surface protein G (SasG). We used anti-Eap antibody and the *spa sbi* double knockout strain of MR23 (MR23 Δ*spa* Δ*sbi*) because staphylococcal protein A (Spa) and second immunoglobulin-binding protein (Sbi) inhibit the specific immunodetection of target proteins (Supplementary Fig. 8a) via interaction with immunoglobulin G (IgG). MR23 Δ*spa* Δ*sbi* forms a robust biofilm equivalent to that of the wild-type strain^33^ and antibodies reached to the bottom of the biofilm and successfully revealed the localization of Eap throughout the whole-biofilm structure (Fig. 3a and Supplementary Movie 11). A considerable amount of Eap was localized in the bottom layer of the biofilm and attached to the surface of the substratum. In contrast, control experiments showed no fluorescence in the absence of anti-Eap primary antibody (Supplementary Fig. 8b), nor when MR23 Δ*spa* Δ*sbi* Δ*eap* was used instead of MR23 Δ*spa* Δ*sbi* (Supplementary Fig. 8c), indicating that Eap was specifically detected by the antibody. In addition, Eap was rarely detected in the upper region of the biofilm (Fig. 3a), suggesting that Eap plays a key role in cell-to-substrate interactions, rather than functioning as a shelter on the surface of the biofilm. A close-up view indicated that Eap was detected as dots or short fibers at the interfaces between *S. aureus* cells (Fig. 3b), suggesting that Eap plays a role in cell-to-cell interactions as a molecular glue. These results are consistent with previous observations that Eap promotes cell aggregation^25^. In addition, the distribution of Eap was distinct from that of the cell wall-anchored protein SasG, which also plays an important role in the formation of thick MR23 biofilms^9^. SasG localized at the surface of only a small number of cells, forming a ring-like shape. (Fig. 3c, d, and Supplementary Movie 12). The ring-like distribution of SasG resembled that of other cell wall-anchored proteins, such as Spa^34,35^, clumping factor A, fibronectin-binding protein B, and serine-aspartate repeat-containing protein C^36^. In addition, SasG-expressing cells formed small clusters (Fig. 3d and Supplementary Movie 12). According to the cell wall-anchoring mechanism of SasG, the surface-localized SasG should be expressed and secreted from each cell that displays it at the surface^37^.

The next question we addressed was why Eap showed such characteristic distribution patterns in the observed biofilms. One possible scenario was that the spatial distribution of Eap-expressing cells determined the localization of the protein. To investigate this, we generated MR23 Δ*spa* Δ*sbi* expressing *eap::mScarlet-1* transcriptional fusion from the chromosome and examined cells expressing Eap using iCBiofilm. As shown in Fig. 3e, Eap-expressing (mScarlet-1 positive) cells were localized to the bottom half of the biofilm. Secreted Eap was detected in a similar region and on the surface of the substratum, as described in the first Eap experiments above. Eap-expressing cells and secreted Eap were rarely observed in the upper region of the biofilm. A close-up view revealed that Eap-expressing cells were distributed heterogeneously and formed clusters in which secreted Eap was predominantly localized at the interface between cells (Fig. 3f). In addition, the number of SasG-expressing cells was lower than that of Eap-expressing cells and subpopulations of Eap-expressing cells co-expressing SasG (Fig. 3g).

These results indicate that iCBiofilm can be used for multicolor imaging of matrix proteins and bacterial cells in biofilms using fluorescence-labeled antibodies, fluorescent proteins, and nucleic acid probes.

### iCBiofilm allows to image the heterogeneous distribution of matrix polysaccharides and eDNA

**Fig. 4:**
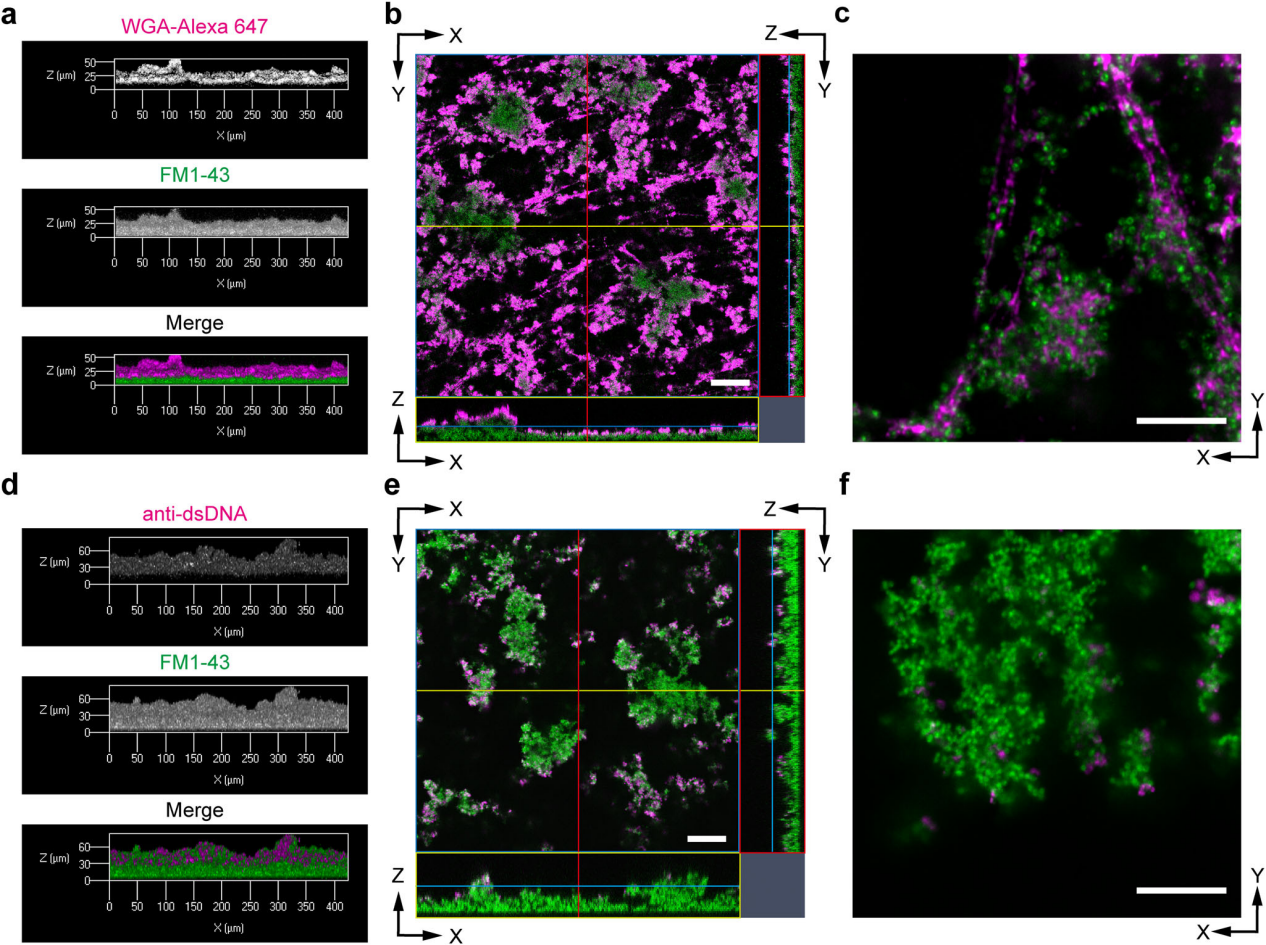
Application of the iCBiofilm method to visualize polysaccharide intercellular adhesin (PIA) and eDNA in *S. aureus* biofilms. **a**–**c** Confocal laser scanning microscopy (CLSM) images of the *S. aureus* MR10 biofilm stained with WGA-Alexa 647 (PIA, magenta) and FM1-43 (cells, green). **d**–**f** CLSM images of the *S. aureus* MR23 Δ*spa* Δ*sbi* biofilm stained with anti-dsDNA mouse monoclonal antibody and Alexa 647-conjugated anti-mouse IgG (eDNA, magenta) and FM1-43 (cells, green). The biofilm was soaked in 35.2% (w/w) iohexol solution. The typical side views of the biofilms (**a**, **d**), orthogonal images (**b**, **e**), and the X-Y sections for the selected z-stack positions (**c**, **f**) acquired using ×20 and ×63 objective lenses are shown. Scale bars are 50 μm in **b**, **e** and 10 μm in **c**, **f**.

Next, we used iCBiofilm to observe extracellular polysaccharides and eDNA in an *S. aureus* biofilm. Alexa Fluor 647-conjugated wheat germ agglutinin (WGA-Alexa 647) was used to detect extracellular polysaccharides in an *S. aureus* biofilm because it binds strongly with poly-N-acetylglucosamines, a major component of staphylococcal extracellular polysaccharides^25,26^. In addition, *S. aureus* MR10, a clinically isolated strain of methicillin-resistant *Staphylococcus aureus*, was selected because it produces a large amount of polysaccharides and forms a robust polysaccharide-dependent biofilm^25,26^. As shown in Fig. 4a, b, an MR10 biofilm formed with a thickness of 25–50 µm and a surface layer of polysaccharides. A high-magnification image highlighted networks of extracellular polysaccharide fibers connecting *S. aureus* cells, suggesting that they functioned as intercellular adhesins (Fig. 4c).

Furthermore, eDNA was detected using the anti-dsDNA monoclonal antibody. The MR23 Δ*spa* Δ*sbi* strain was used because it lacks IgG binding proteins, which interfere with specific detection of proteins using antibodies^33^. eDNA was detected mainly in the upper region of the biofilm (Fig. 4d, e). In contrast to the fibrillar structures of extracellular polysaccharides (Fig. 4c), eDNA was detected as dots and aggregates on bacterial cell surfaces (Fig. 4f).

Taken together, these findings indicate that iCBiofilm is useful for imaging the heterogeneous distribution of multiple biofilm matrix components in thick biofilms of *S. aureus*, and for analyzing the mechanisms of how such patterns are determined.

### Imaging of neutrophils and phagocytosis in *S. aureus* biofilms using iCBiofilm

**Fig. 5:**
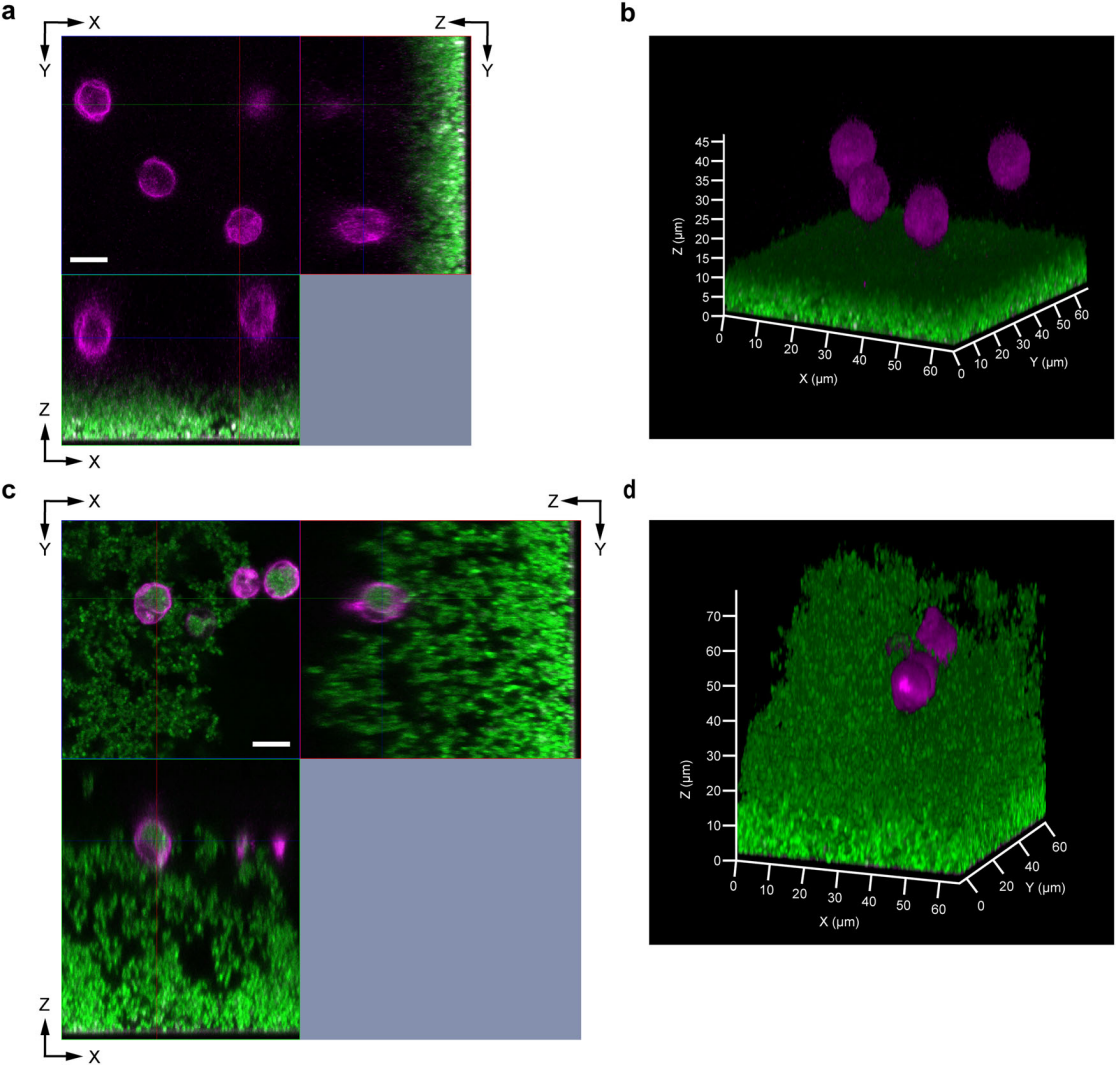
iCBiofilm imaging for neutrophil phagocytosis of *S. aureus* biofilm cells. Biofilms of *S. aureus* MR23 and neutrophils from mice were stained with FM1-43 (green) and Alexa 647-conjugated anti-Ly-6G (magenta), respectively. Neutrophils were also stained with FM1-43 but can be distinguished from bacterial cells because of their size and shape. Large magenta shapes represent neutrophils, while green shapes represent biofilm cells. The specimens were soaked in PBS (**a**, **b**) or 37.1% (w/v) ioversol (**c**, **d**) and observed using an LSM880 microscope with a ×63 objective lens and an Airyscan super-resolution unit. **a**, **c** Orthogonal images. Scale bars are 10 μm. **b**, **d** 3D images.

Bacterial cells in a biofilm are believed to be protected from host defense systems, such as neutrophils and antimicrobial peptides^1^. We aimed to examine this using iCBiofilm. Here, ioversol was used instead of iohexol because iohexol tended to disperse non-fixed *S. aureus* biofilms (Supplementary Fig. 1) and may dissociate the interaction between biofilms and neutrophils. As shown in Fig. 5a, b, and Supplementary Fig. 9a, neutrophils appeared to float above the biofilm, which may falsely suggest the existence of non-visible matrix components at the interface between visualized neutrophils and the biofilm. However, iCBiofilm imaging clearly demonstrated that neutrophils penetrated into the thick biofilm (Fig. 5c, d, Supplementary Fig. 9b) and phagocytosed the *S. aureus* cells (Fig. 5c and Supplementary Fig. 9c), which is inconsistent with the long-established notion that biofilm-embedded cells are protected from neutrophil phagocytosis by the biofilm matrix. Our results imply a concept that some cells in the biofilm can work as decoys, trapping neutrophils away from the other biofilm cells. This strategy might contribute to the survival of biofilm cells encountering phagocytotic cells. These observations suggest that iCBiofilm could be useful to investigate the interactions between biofilms and the host immune defenses.

### Imaging of Gram-negative bacterial biofilm using iCBiofilm

**Fig. 6:**
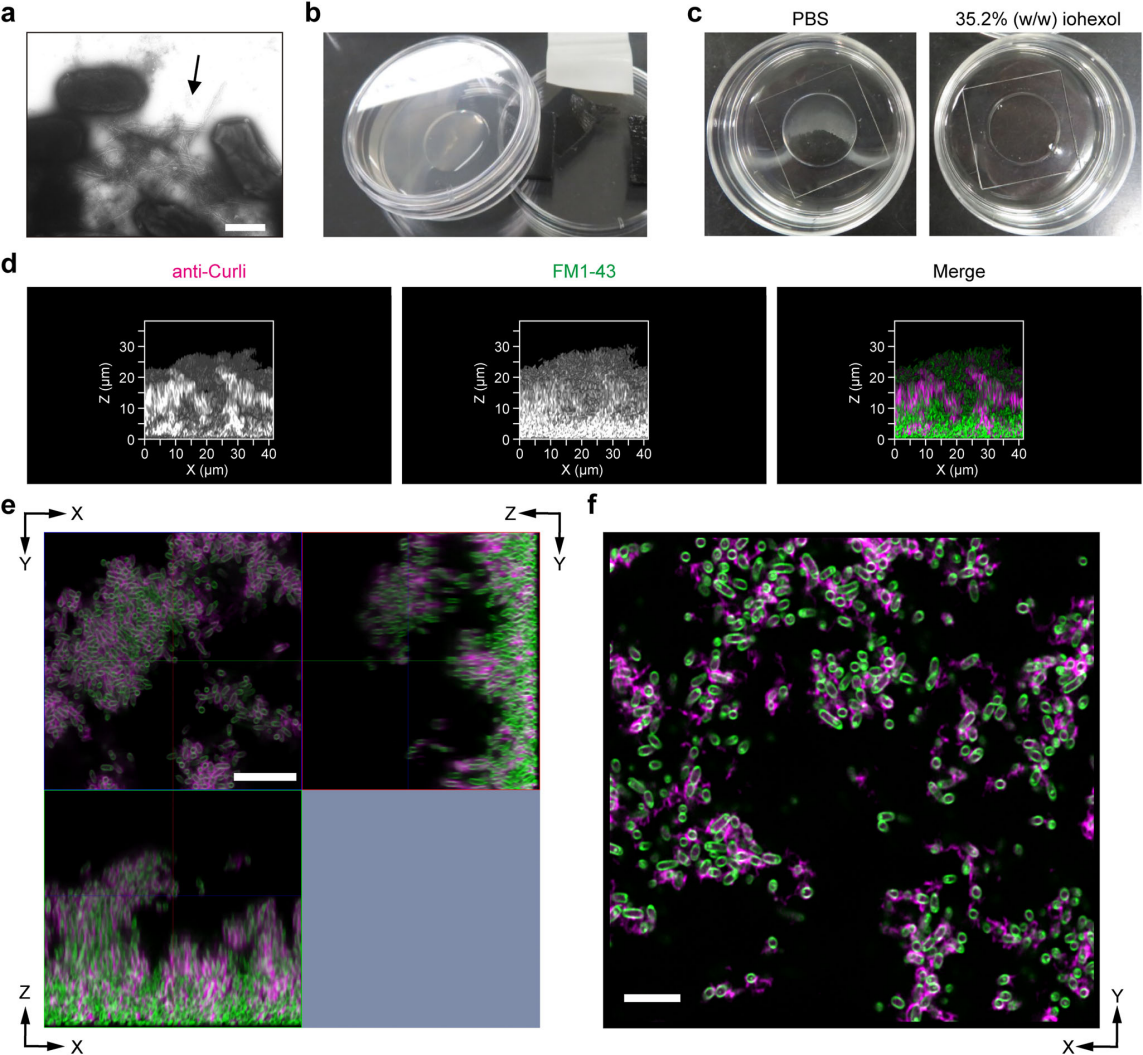
Application of the iCBiofilm method to Gram-negative bacterial biofilm. **a** A typical transmission electron microscopy image of *E. coli* BW25113 grown on YESCA agar plates. The image shows bacterial cells surrounded by Curli amyloid fibers (black arrow). **b** Image of slanted YESCA liquid culture to form *E. coli* biofilm at the air-liquid interface. **c** *E. coli* biofilms soaked in PBS or 35.2% (w/w) iohexol. **d** Typical side views of *E. coli* biofilms stained with anti-Curli rabbit IgG and Alexa 647-conjugated anti-rabbit IgG (Curli, magenta) and FM1-43 (cells, green). The biofilm was soaked in 35.2% (w/w) iohexol solution and observed using an LSM800 microscope with ×63 and ×100 objective lenses and an Airyscan super-resolution unit. **e** Orthogonal images. **f** A typical X-Y section for the selected z-stack position. Scale bars are 500 nm in **a**, 10 μm in **e**, and 5 μm in **f**.

To expand the applicability of our method to imaging a gram-negative biofilm, we used iCBiofilm to observe an *Escherichia coli* biofilm, aiming identify Curli proteins, extracellular amyloid fibers produced by such enterobacteria. A previous study using fluorescence and scanning electron microscopy revealed the microanatomy and microphysiology of an *E. coli* macrocolony biofilm formed on agar plates at an unprecedented cellular resolution^38^; however, little is known about aqueous *E. coli* biofilms. In this study, we cultivated *E. coli* BW25113 in YESCA at 25 °C for 3–7 days, thereby producing Curli proteins (Fig. 6a) within a robust biofilm at the air–liquid interface (Fig. 6b, c)^39,40^. As in the case of *S. aureus* biofilms, the *E. coli* biofilm was opaque and became transparent after soaking in 35.2% (w/w) iohexol (Fig. 6c). Indirect immunofluorescence microscopy using an anti-Curli antibody showed the distribution of Curli proteins throughout the aqueous biofilm (Fig. 6d, e). A close-up view revealed the existence of fibrillar structures stained with the anti-Curli antibody, and cells were encased in a network of Curli fibers (Fig. 6f).

A previous study has found that Curli and Curli-expressing cells localize in the outer region of microcolonies formed on agar plates, whereas the bottom zones of these biofilms feature elongated dividing cells and a tight mesh of entangled flagella^41^. This heterogenous distribution of Curli was inconsistent with the observations presented in this study (Fig. 6d, e). These results indicate that the characteristics of *E. coli* biofilms and the distribution of Curli fibers depend on the culture conditions. These differences may explain the distinct susceptibility of aqueous biofilms and microcolonies of Curli-producing *E. coli* to antibiofilm agents such as green tea catechin, epigallocatechin-3-gallate (EGCG)^39,41^ and the plant-derived flavonoid myricetin^42,43^. For instance, EGCG promoted the degradation of RNA polymerase sigma factor S (RpoS), one of the master regulators for Curli biosynthesis, via degradation by the ClpXP ATP-dependent protease in aqueous biofilms^39^, but did not affect the cellular level of RpoS in microcolonies^41^.

### Imaging of fungal biofilm using iCBiofilm

**Fig. 7:**
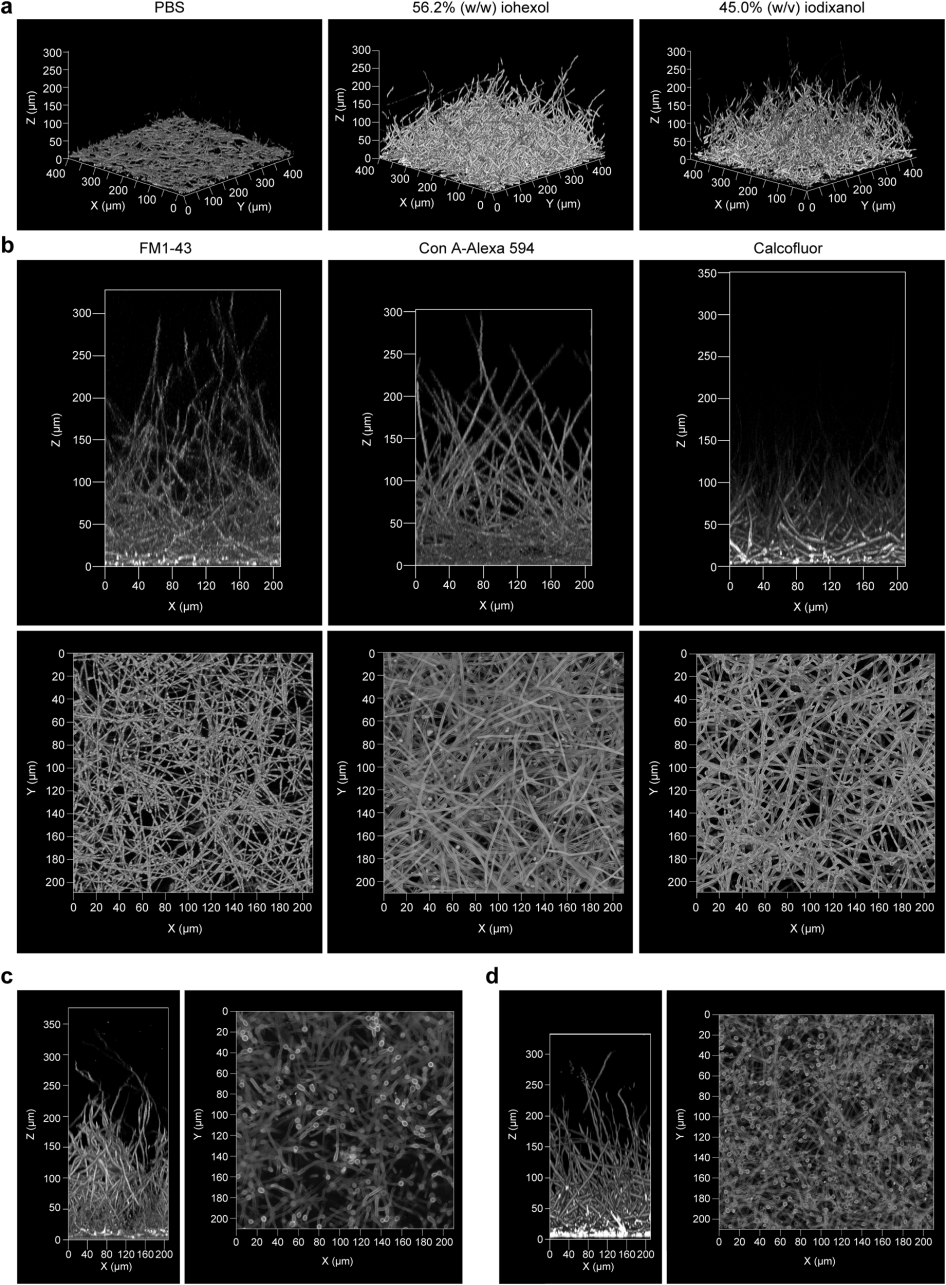
Application of the iCBiofilm method to a *C. albicans* biofilm. **a** 3D images of *C. albicans* biofilms stained with FM1-43. The stained biofilms were soaked in the indicated solutions and observed using an LSM880 microscope with a ×20 objective lens. **b** Side and bottom views of *C. albicans* biofilms stained with indicated dyes. **c** Side and bottom views of *C. albicans* biofilms formed under the conditions employed in the Douglas model^45^. **d** Side and bottom views of *C. albicans* biofilms grown under similar conditions in **a** and **b**, but with an initial number of *C. albicans* cells of 10^7^ CFU/mL. The biofilms were stained with Con A-Alexa 594, soaked in 45.0% (w/v) iodixanol, and observed using the LSM880 microscope.

To test the applicability of iCBiofilm to fungal biofilms, we chose *Candida albicans* as a model major pathogenic fungus that causes biofilm-associated infections in humans. However, three-dimensional imaging of whole *C. albicans* biofilms with a thickness of more than 100 µm is conventionally challenging^44^. As shown in Fig. 7a, only the bottom region of the biofilm was observed in the PBS control, whereas the entire biofilm was observed using 56.2% (w/w) iohexol. In addition, 45.0% (w/v) iodixanol was also effective for visualizing the *C. albicans* biofilm (Fig. 7a), indicating that RI-matching media are sufficient for making *C. albicans* biofilms transparent and allowing deep imaging.

Next, iodixanol was compared to commercially available mounting reagents. Iodixanol was superior to the other media for visualizing *C. albicans* biofilms more than 500 μm in thickness (Supplementary Fig. 10a). In addition, some immersion media, such as Super Clear Mount, Immersion M, ProLong Diamond, and SlowFade Diamond, partially destroyed the paraformaldehyde (PFA)-fixed *C. albicans* biofilm (Supplementary Fig. 10b) or the *S. aureus* biofilm (Supplementary Fig. 10c). Based on these findings, we recommend 15.0-45.0% (w/v) iodixanol or 35.2–56.2% (w/w) iohexol for the imaging of *C. albicans* and *S. aureus* biofilms.

For staining the thick *C. albicans* biofilm, Alexa Fluor 594-conjugated concanavalin A (Con A-Alexa 594) was deemed more appropriate than FM1-43 and Calcofluor White (Fig. 7b). Specifically, the use of Calcofluor White is not recommended for future studies, as it only allowed us to visualize a basal region of the biofilm due to short excitation and emission wavelengths.

Bottom views of the biofilm revealed many pseudohyphae and hyphae, but only a small number of yeast-form cells (Fig. 7b), a result inconsistent with the Douglass model. In this model, it is proposed that yeast-form cells attach to the substrate and form a basal polylayer that functions to anchor the biofilm. This is followed by germ tube appearance from upper yeast-form cells, hyphae elongation, and deposition of an extracellular matrix composed of proteins, carbohydrates, lipids, and nucleic acids^45^. This discrepancy could be due to differences in the culture conditions. Therefore, we prepared a *C. albicans* biofilm according to the procedure of the Douglas model. Although a higher number of yeast-form cells was observed at the bottom of the biofilm compared to the previous procedure, they did not form a polylayer (Fig. 7c). A similar result was observed when the number of initial *C. albicans* cells increased from 10^6^ to 10^7^ CFU/mL (Fig. 7d). We believe that our iCBiofilm procedure considerably alleviated the limitation of laser penetration into thick biofilms using conventional upright CLSM and improved the imaging of *C. albicans* biofilms, especially at the bottom region of the 3-D structure.

**Fig. 8:**
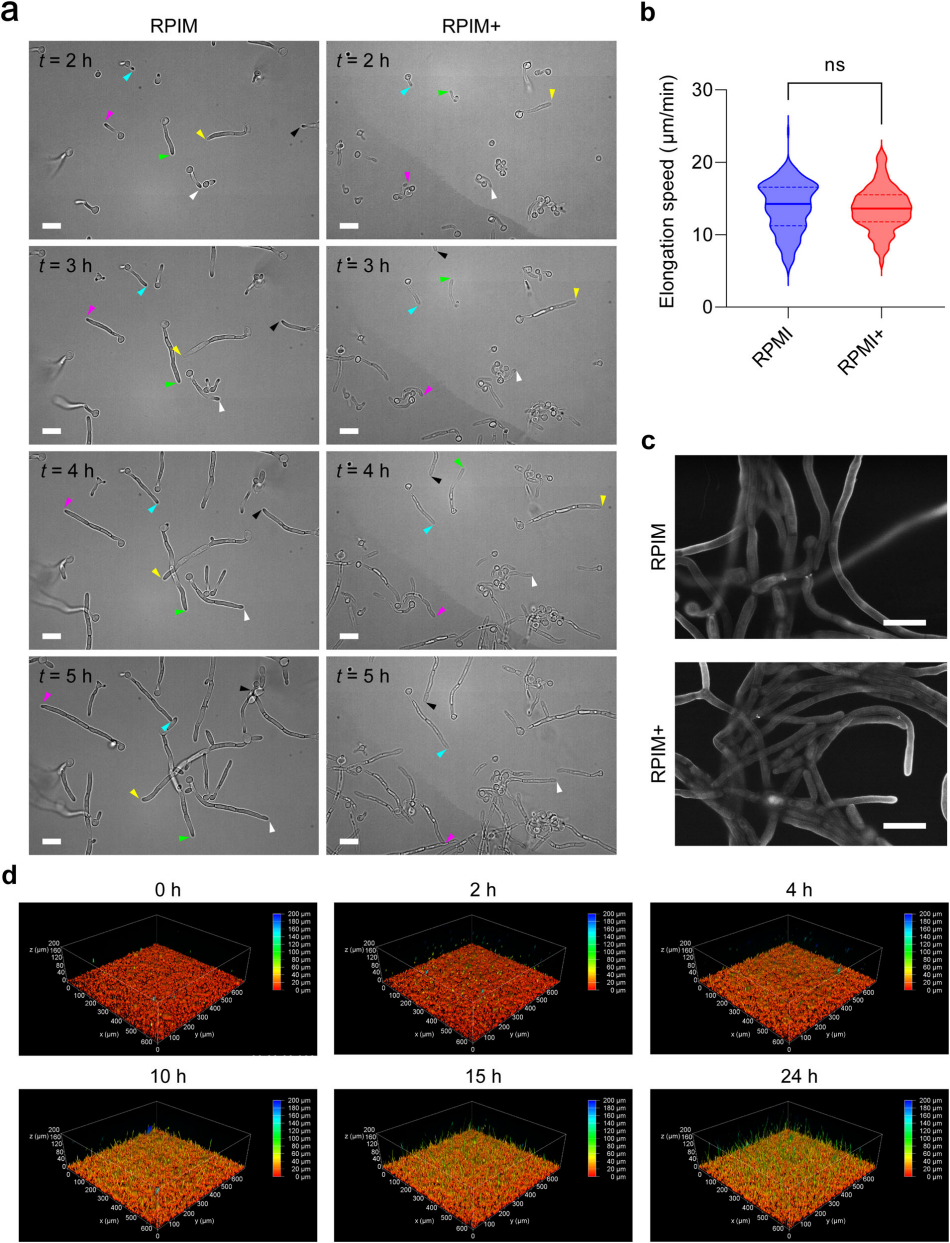
Live-cell clearing imaging for biofilm formation of *C. albicans*. **a** Growth of *C. albicans* SC5314 cells on RPMI agar plates in the absence (RPMI) and presence of 30.0% (w/v) iodixanol (RPMI+) were observed by phase-contrast microscopy. Colored arrowheads indicate individual elongating hyphae. **b** Violin plots of the elongation speeds of hyphae analyzed using microscopic images taken every 1 min. RPMI, *n* = 235. RPMI+, *n* = 185. ns, not significant (unpaired two-tailed Student *t* test). **c** ASEM images of SC5314 cells grown in RPMI and RPMI + liquid cultures. **d** The biofilm of SC5314 was formed in RPMI-MOPS (pH 7.0) containing 24% (w/v) iodixanol and 1-μM MitoTracker Deep Red in a glass-bottomed dish. 3D images at the indicated time points are shown in a heat map color scale representing the thickness of the biofilm. Scales are 10 μm in **a**, **c**.

Furthermore, biofilm formation by *C. albicans* was visualized by live cell imaging using iCBiofilm and the THUNDER imaging system, since iodixanol did not affect cell growth (elongation rate of hyphae) and shape of *C. albicans* (Fig. 8a–c). In the initial 3 h, a basal layer of the biofilm with a thickness of approximately 20 μm and many falling planktonic cells was observed (Fig. 8d and Supplementary Movie 13). Subsequently, germination and elongation of hyphae were clearly visualized 4 h after onset (Fig. 8d and Supplementary Movie 13). Elongation of hyphae continued over time, and after 24 h, the thickness of the biofilm reached approximately 200 μm (Supplementary Movie 13). Single-cell analysis for *C. albicans* cells existing a basal layer demonstrated again that many yeast-form cells attached to the glass surface followed by germination and elongation of hyphae in several hours after inoculation (Supplementary Fig. 11a). Elongation rates of true hyphae and pseudohyphae were similar (Supplementary Fig. 11b, c).

To our knowledge, this is the first study to visualize the development of a living biofilm with a thickness of >100 μm at a single-cell resolution.

## Conclusions

This study aimed to develop an optical clearing method for investigating biofilms. The resulting method, iCBiofilm shows promise as a tool for deep imaging of various microbial biofilms in vitro. Compared with conventional tissue-clearing methods (CUBIC, Sca*l*eS, and SeeDB2), iCBiofilm is a rapid, simple, cost-effective, and non-invasive method, as it only requires soaking the biofilms in RI-matching media (Fig. 1 and Table 1). iCBiofilm allows live cell imaging of several microbial biofilms (*S. aureus*, *S. epidermidis*, and *C. albicans*), a feat previously considered difficult using conventional tissue clearing.

The finally adapted iCBiofilm procedures are described in Supplementary Fig. 12 and the subsection of “Clearing of fixed biofilms with iCBiofilm” in Methods. For live-cell imaging of biofilms, it is recommended to add iodixanol in a suitable culture broth at 15–30% (w/v). Biofilm formation of bacteria and fungi constitutively expressing fluorescent proteins may be imaged over time without staining any fluorescent probes. Alternatively, ThT and Mitotracker reagents can be used for live-cell imaging. For 3D biofilm imaging, biofilms should be fixed with 4% PFA or 1% GA and 4% PFA and then strained with one or more suitable fluorescent probes. The choice of the fixing reagents and fluorescent probes depend on properties of interested biofilms and experimental purposes. Opaque biofilms can be cleared immediately by adding one of the iCBiofilm regents and imaged by CLSM or other optical microscopies. As the first choice, 45% (w/v) iodixanol is recommended to use for clearing of fragile biofilms, while 35.2% (w/w) iohexol is recommended for stable biofilms. As shown in Supplementary Fig. 2, iohexol is replacebale with other RI-matching media including iodixanol, ioversol, iopamidol, and iopromide, but iohexol is capable of more rapid clearing compared with the others. Diatrizoate is not recommended for biofilm clearing, since it decolorized the stained *S. aureus* biofilm. If clearing is not sufficient, it is recommended to optimize the concentration of the RI-matching media for individual biofilms.

Our approach nevertheless shows some limitations. The concentrations of iodixanol used for live cell imaging in this study were slightly lower than the optimum concentration for biofilm clearing, as higher concentrations inhibited biofilm formation of the tested microorganisms on the glass surface. Therefore, to achieve the highest imaging quality and optimum conditions for biofilm formation of various microorganisms, alternative RI-matching media should be developed in the future. Since low-viscosity iodixanol was superior to high-viscosity iohexol for live-cell imaging of biofilm development, we believe that the viscosity of RI-matching media is a key point for screening alternative RI-matching media.

The iCBiofilm method was also useful for imaging interactions between biofilms and host defense systems. We were able to analyze phagocytosis of biofilm cells by neutrophils. However, we fixed the specimens using PFA; therefore, the obtained images were snapshots of phagocytosis. Live-cell imaging of biofilm phagocytosis by neutrophils is of great interest but remains a challenge because a technique for the preparation of fully active neutrophils labeled with fluorescent or genetically modified probes to stably express fluorescent proteins has not been developed. In addition, favorable conditions for biofilm growth differ from those for neutrophils. When these technical issues are resolved, iCBiofilm may be used to perform live cell imaging of biofilm-neutrophil interactions.

Moreover, all biofilms imaged here were formed under in vitro static conditions. Future experiments should focus on determining the fine structures of biofilms formed in vivo (e.g., biofilms formed on the surfaces of medical devices and tissues) and semi in vivo conditions (using a flow cell system), and in natural environments. Our findings can therefore contribute to the advancement of biofilm research and be used as a tool to identify or qualify antibiofilm approaches in clinical and industrial settings.

## Methods

### Microbial strains and media

The *S. aureus*, *S. epidermidis*, *E. coli*, and *C. albicans* strains used in this study are listed in Supplementary Table 1. *E. coli* was grown in Lysogeny broth (LB) (1% tryptone, 0.5% yeast extract, 1% NaCl) (Kanto Chemical Co., Inc., Tokyo, Japan) and YESCA (1% casamino acids, 0.1% yeast extract). *S. aureus* and *S. epidermidis* were grown in BHI medium (Becton Dickinson, Franklin Lakes, NJ, USA). These bacteria were cultured at 37 °C unless otherwise noted. If required, the medium was supplemented with 1% glucose to promote biofilm formation or with antibiotics to select transformants.

*C. albicans* was grown on a yeast-peptone-dextrose (YPD) plate (1% yeast extract, 2% peptone, 2% dextrose, and 2% agar), RPMI 1640 (Gibco; Thermo Fisher Scientific, Waltham, MA, USA) buffered with MOPS (pH 7.0) (RPMI-MOPS), or sLee’s medium [0.5% NaCl (FUJIFILM Wako Chemicals, Tokyo, Japan), 0.5% ammonium sulfate (FUJIFILM Wako Chemicals), 0.25% potassium phosphate dibasic (Nacalai Tesque, Kyoto, Japan), 0.13% l-leucine (Nacalai Tesque), 0.1% l-lysine (Kanto Chemical Co.), 0.05% l-alanine (FUJIFILM Wako Chemicals), 0.05% l-phenylalanine (FUJIFILM Wako Chemicals), 0.05% l-proline (FUJIFILM Wako Chemicals), 0.05% l-threonine (Kanto Chemical Co.), 0.02% magnesium sulfate (FUJIFILM Wako Chemicals), 0.01% l-methionine (FUJIFILM Wako Chemicals), 0.007% l-ornithine (Nacalai Tesque), 0.007% l-arginine (FUJIFILM Wako Chemicals), 0.0001% D-biotin (Nacalai Tesque), 0.1 mM zinc sulfate (FUJIFILM Wako Chemicals), and 1.25% dextrose (FUJIFILM Wako Chemicals)].

### Biofilm-clearing reagents

SeeDB2 Trial Kit, SCALEVIEW-S Trial Kit, deScale Solution, and CUBIC Trial Kit were purchased from FUJIFILM Wako Chemicals. SeeDB2 Trial Kit contained SeeDB2G [56.2% (w/w) iohexol, 10 mM Tris-HCl (pH 7.6), and 0.267 mM EDTA]^24^, SeeDB2S [70.4% (w/w) iohexol, 10 mM Tris-HCl (pH 7.6), and 0.267 mM EDTA]^24^, saponin (powder), and sterilized solution of PBS (-). SCALVEIW-S Trial Kit contained SCALEVIEW-S0, SCALEVIEW-S1, SCALEVIEW-S2, SCALEVIEW-S3, SCALEVIEW-S4, and SCALEVIEW-SMt solutions. CUBIC Trail Kit contained Sca*l*eCUBIC-1 Solution, Sca*l*eCUBIC-2 Solution, Mounting Solution 1, and Mounting Solution 2. Iohexol [Omnipark 350; 56.2% (w/w)] was purchased from Daiichi Sankyo (Tokyo, Japan). Iodixanol [Optiprep; 60.0% (w/v)] was purchased from Thermo Fisher Scientific. Ioversol [Optiray 350; 74.1% (w/v)] was purchased from Guerbet (Villepinte, France). Iopromide [Iopromide 370; 76.9% (w/v)], Iopamidol [Iopamidol 370; 75.6% (w/v)], and diatrizoate [Urografin 60%; 47.2% (w/v)] were purchased from Fujifilm Wako Chemicals, Hikari Pharmaceutical Co. (Tokyo, Japan), and Bayel (Tokyo, Japan), respectively. When required, the reagents were diluted with double-distilled water (DDW).

### Fluorescent reagents

Con A-Alexa 594, WGA-Alexa 647, NucBlue Fixed Cell ReadyProbe Reagent (DAPI), FilmTracer FM 1–43 Green Biofilm Cell stain (FM1-43), MitoTracker Deep Red FM, and the FilmTracer LIVE/DEAD Biofilm Viability Kit were purchased from Thermo Fisher Scientific. Calcofluor white and ThT were purchased from Sigma-Aldrich (St. Louis, MO, USA) and AAT Bioquest (Sunnyvale, CA, USA), respectively.

### Antibodies

Rabbit anti-Eap polyclonal antibody was developed by Scrum (Tokyo, Japan)^25^. Rabbit anti-SasG polyclonal antibody^9^ and mouse anti-SasG polyclonal antibodies were developed by Eurofins Genomics (Tokyo, Japan). Rabbit anti-Curli antibody was developed by Scrum (Tokyo, Japan), and mouse anti-dsDNA monoclonal antibody was purchased from Abcam (Cambridge, MA, USA)^33^. Alexa 405-conjugated anti-mouse IgG, Alexa 488-conjugated goat anti-rabbit IgG, Alexa 647-conjugated goat anti-rabbit IgG, and Alexa 647-conjugated goat anti-mouse IgG were purchased from Thermo Fisher Scientific. Alexa Fluor 647-conjugated anti-mouse lymphocyte antigen 6 complex locus G antibody (anti-Ly-6G-Alexa 647) was purchased from BioLegend Japan, Inc. (Tokyo, Japan).

### Plasmid and strain construction

To construct the Eap-mScarlet-1 transcriptional fusion in *S. aureus* MR23, a DNA fragment containing the 3’-end 500-bp fragment of MR23 *eap*, a ribosome-binding site, the mScarlet-1 gene with its codon optimized for *S. aureus*, and the 500-bp fragment of the downstream region of MR23 *eap* (eap-mScarlet-1) was generated and cloned into pEX-K4J2 by Eurofins (Tokyo, Japan). The resulting plasmid was named pEX-eap-mS1 (Supplementary Table 1). The inserted DNA was amplified using pEX-eap-mS1 as a template, KOD Plus Neo DNA polymerase (Toyobo, Osaka, Japan), and the primers eap-mScarlet-pKOR1-F and eap-mScarlet-pKOR1-R (Supplementary Table 2). The amplified DNA was purified with QIAquick DNA purification Kit (Qiagen, Hilden, Germany) and cloned into pKOR1^46^ using the BP Clonase Enzyme Mix (Life Technologies, Palo Alto, CA, USA) according to manufacturer instructions. The resulting plasmid was named pKOR1-eap-mS1 (Supplementary Table 1).

To construct the SasG-mNeonGreen transcriptional fusion in *S. aureus* MR23, a DNA fragment containing the 3’-end 500-bp fragment of MR23 *sasG*, a ribosome-binding site, the mNeonGreen gene with its codon optimized for *S. aureus*, and the 500-bp fragment of the downstream region of MR23 *sasG* was generated and cloned into pEX-K4J2 by Eurofins (Tokyo, Japan). The resulting plasmid was named pEX-sasG-mNG (Supplementary Table 1). The inserted DNA was amplified using pEX-sasG-mNG as a template, KOD Plus Neo DNA polymerase (Toyobo, Osaka, Japan), and the primers sasG-mNeon-pKOR1-F and sasG-mNeon-pKOR1-R (Supplementary Table 2). The amplified DNA was cloned into pKOR1^46^ using the BP Clonase Enzyme Mix according to manufacturer instructions. The resulting plasmid was named pKOR1-sasG-mNG (Supplementary Table 1).

Using pKOR1-eap-mS1 and pKOR1-sasG-mNG, allelic exchange was performed^9,33^. Briefly, these plasmids were transformed into *S. aureus* RN4220 by electroporation^47^. The plasmids purified from RN4220 were further transformed into the target strain MR23 Δ*spa* Δ*sbi* by electroporation, and the mScarlet-1 and mNeonGreen genes were inserted into the genome by homologous recombination^46^. The constructed strains were named MR23 Δ*spa* Δ*sbi eap-mS1*, MR23 Δ*spa* Δ*sbi sasG-mNG*, and MR23 Δ*spa* Δ*sbi eap-mS1 sasG-mNG* (Supplementary Table 1).

### Biofilm formation

Biofilm formation in 96-well polystyrene plates was performed^9,33^. *S. aureus* strains MR4 and MR23 and *S. epidermidis* strain SE21 were grown in BHI (2 mL) at 37 °C for 16–20 h. The cultures were diluted 1:1000 in BHIG and the suspensions (200-μL) were cultured at 37 °C for 24 h in 96-well polystyrene flat-bottom plates (Corning, Corning, NY). The resulting biofilms were used to examine the effects of clearing reagents on optical density and stability.

### Optical density measurement and biofilm stability test

After biofilm formation in 96-well polystyrene plates as described above, culture media and planktonic bacterial cells were removed and clearing reagent (100 μL) at the indicated concentrations (See “Biofilm clearing reagents”) or PBS was added to the biofilms. To ensure experimental accuracy, at least three wells were used per reagent. Subsequently, optical densities at 595 nm and 492 nm were measured using an Infinite F200 Pro microplate reader (Tecan, Männedorf, Switzerland). Clearing reagents were then removed and biofilms were carefully washed once with PBS (100 μL). Residual biofilms were stained with 0.05% crystal violet (FUJIFILM Wako Chemicals) (50–100 μL) for 10 min at 25 °C. After staining, each biofilm was washed once with 100 μL of PBS and its mass was quantified by measuring the absorbance at 595 nm using the Infinite F200 Pro microplate reader.

### Fluorescence staining of biofilms for CLSM

Overnight cultures of *S. aureus* strains grown in BHI at 37 °C for 16–20 h were diluted 1:1000 in BHIG and incubated at 37 °C for 24 h in glass-bottomed dishes (35-mm diameter; Matsunami Glass, Osaka, Japan). To test the effect of iohexol on biofilm formation, *S. aureus* biofilms were formed in BHIG containing 28.1% (w/w) iohexol at 37 °C for 24 h in a glass-bottomed dish. Biofilms were fixed with 1% (w/v) GA, 4% PFA, or both in PBS for 30 min at 25 °C. After the removal of the fixing solutions, biofilms were washed three times with DDW, and the fixed biofilms were stained with fluorescent probes or antibodies.

For fluorescent staining of *S. aureus* cells, biofilms were stained with 25 μM ThT, or 5 μg/mL FM1-43, which stains bacterial membranes, for at least 30 min at 25 °C. When required, biofilms were stained with DAPI, which stains DNA in fixed cells. For the identification of extracellular polysaccharides, *S. aureus* biofilms were stained with WGA-Alexa 647 at 25 °C for more than 3 h or at 4 °C overnight.

For immunofluorescence staining, *S. aureus* MR23 Δ*spa* Δ*sbi*^33^ was used. In addition, the derivatives Δ*spa* Δ*sbi* Δ*eap*, Δ*spa* Δ*sbi eap-mS1*, MR23 Δ*spa* Δ*sbi sasG-mNG*, and Δ*spa* Δ*sbi eap-mS1 sasG-mNG* were used. PFA-fixed *S. aureus* biofilms were incubated in blocking buffer [3% bovine serum albumin (BSA), 0.05% Triton X-100, PBS, pH 7.4] for 30 min at 25 °C. Subsequently, the biofilms were incubated with anti-Eap rabbit antibody or anti-SasG rabbit antibody (diluted 1:200 in blocking buffer). After incubation for 3 h at 25 °C, the biofilms were washed three times with blocking buffer and incubated with Alexa 647-conjugated goat anti-rabbit IgG or Alexa 488-conjugated goat anti-rabbit IgG (diluted 1:200 in blocking buffer), respectively, at 4 °C overnight. The biofilms were washed three times with PBS and fixed with 1% GA and 4% PFA in PBS for 30 min at 25 °C. Biofilm cells were then stained with 5 μg/mL FM1-43 for at least 30 min at 25 °C or overnight at 4 °C. For the detection of eDNA, mouse anti-dsDNA monoclonal antibody and Alexa 647-conjugated goat anti-mouse IgG antibody were used following the same procedure.

Overnight cultures of *E. coli* BW25113 grown in LB at 30 °C were diluted 1:1000 in YESCA and grown at 25 °C for 5 days in glass-bottomed dishes (35-mm diameter; Matsunami Glass). The dishes were placed on a slant to form an air-liquid interface conducive to *E. coli* biofilm formation^40^. The resulting biofilms were fixed in 4% PFA in PBS for 30 min at 25 °C. After removal of the fixing solution, biofilms were washed three times with PBS and incubated in blocking buffer (3% BSA, 0.05% Triton X-100, PBS, pH 7.4) at 25 °C for 1 h. Then, the biofilms were incubated with anti-Curli antibody (diluted 1:100 in blocking buffer) at 25 °C for 3 h or left untreated as a control. After washing three times with blocking buffer, the biofilms were further incubated with Alexa 647-conjugated goat anti-rabbit IgG (diluted 1:100 in blocking solution) at 4 °C overnight. After washing three times with PBS, the biofilms and antibodies were fixed with 1% GA and 4% PFA in PBS at 25 °C for 30 min, washed three times with PBS, and stored in PBS containing 10 μg/mL FM1-43 at 4 °C until use.

*C. albicans* SC5314 was grown on YPD plates at 25 °C for 3 days, then suspended in RPMI-MOPS. Two milliliter samples containing approximately 10^6^–10^7^ CFU/mL of *C. albicans* cells were grown at 37 °C for 48 h in 35 mm glass-bottomed dishes (Matsunami Glass). A *C. albicans* biofilm was also formed according to the Douglas model^48^ with slight modifications. Briefly, planktonic cells of *C. albicans* SC5314 were grown in 5 mL of Lee’s medium in a test tube at 37 °C for 48 h with shaking at 150 rpm, then diluted in RPMI-MOPS to 10^7^ CFU/mL. The planktonic cell suspension was added to a 35 mm glass-bottomed dish and incubated statically at 37 °C for 1.5 h to allow adherence to the glass surface. After removal of non-adherent cells and medium replacement, cells were incubated at 37 °C for 1.5 h with gentle rocking (10° deflection, 18 cycles/min). The dish was washed once with 1 mL RPMI-MOPS to remove non-adherent and weakly adherent cells. The residual adherent cells were incubated in 3 mL RPMI-MOPS at 37 °C for 48 h to form a thick biofilm. The resulting biofilm was fixed with PBS containing 1% GA and 4% PFA for 30 min at 25 °C, washed three times with DDW, and stained with either FM1-43 (5 μg/mL in PBS), Con A-Alexa 594 (25 μg/mL in PBS), or Calcofluor White (1 mg/mL) in the dark at 4 °C until use.

After removal of fluorescent dyes, biofilms were soaked in the indicated clearing reagents, mounting solutions, or PBS as a control and visualized using a CLSM microscope.

### Clearing of fixed biofilms with iCBiofilm

Biofilms were formed in BHIG at 37 °C for 24 h in 35-mm glass-bottomed dishes and fixed with PBS containing 4% PFA at 25 °C for 30 min. If biofilms are fragile, fixation with 4% PFA and 1% GA is recommended. After three times washing with PBS, the biofilms were stained with fluorescent probes (FM1-43, fluorescent-labeled antibodies, fluorescent-labeled lectins, etc.). The stained biofilms were soaked in one of the RI-matching media dissolved in DDW. As the first choice, 35.2% (w/w) iohexol is recommended to use for clearing biofilms, since it was applicable to wide range of biofilms and enabled very fast clearing. Of note, the RI-matching medium can be flexibly changed. If clearing is insufficient, its concentration should be optimized for biofilms to be analyzed. In addition, if fixed biofilms are fragile and damaged by iohexol, 45% (w/v) iodixanol is recommended to use instead of iohexol. Finally, the specimens were imaged by CLSM as described below.

### Clearing of fixed biofilms with SeeDB2

SeeDB2 Trial Kit (FUJIFILM Wako Chemicals) was used to conduct biofilm clearing, according to the manufacturer instructions. Briefly, the PFA-fixed biofilm of *S. aureus* MR23 was permeabilized with permeabilization solution composed of PBS (pH 7.4) and 2% (w/v) saponin at 25 °C overnight. Then, the biofilm was cleared by incubating with clearing solution 1 [2% (w/v) saponin, 1/3 SeeDB2G solution, and 2/3 DDW] at 25 °C for 24 h, clearing solution 2 [2% (w/v) saponin, 1/2 SeeDB2G solution, and 1/2 DDW] at 25 °C for 6 h, clearing solution 3 [2% (w/v) saponin and SeeDB2G solution] at 25 °C for 24 h, and then clearing solution 4 [2% (w/v) saponin and SeeDB2S solution supplemented with 25 μM ThT] at 25 °C for 24 h. Finally, the biofilm was soaked in SeeDB2S solution supplemented with 25 μM ThT and examined by CLSM.

### Clearing of fixed biofilms with other tissue-clearing methods

The PFA-fixed biofilms of *S. aureus* MR23 were cleared by other tissue-clearing methods as described in Supplementary Note 1.

### Mice

C57BL/6 J mice (CLEA Japan, Inc., Tokyo, Japan) were maintained under specific-pathogen-free conditions at the Jikei University School of Medicine, Japan.

### Ethical statement

All animal experiments were approved by the ethics committee of the Jikei University School of Medicine, Japan, and were performed in accordance with approved guidelines and regulations.

### Isolation of neutrophils from mice

Six C57BL/6 J mice (males and females mice between 8 and 12 weeks of age) were euthanized following the animal care committee-approved protocol of the institution, and bone marrow cells were harvested from femurs. PBS containing 0.1% BSA was used to flush the femurs. Bone marrow cells were pelleted by centrifugation at 1,500 rpm for 5 min at 4 °C and suspended in 1 mL of ACK Lysing Buffer (Thermo Fisher Scientific) at 25 °C for 2 min to lyse the red blood cells. After adding 9 mL of PBS containing 0.1% BSA, the cells were pelleted by centrifugation at 1,500 rpm for 5 min at 4 °C. The resulting centrifugate was suspended in 6 mL PBS and layered onto 4 mL Histopaque-1077 and 4 mL Histopaque-1119 (Sigma). The cells were then centrifuged at 700 × *g*, without acceleration or deceleration, for 30 min at 25 °C. Neutrophils were collected from the interface between Histopaque-1077 and Histopaque-1119 and washed once with PBS containing 0.1% BSA. Finally, neutrophils were suspended in RPMI 1640 medium and the cell number was counted under a microscope.

### Interaction between *S. aureus* biofilm and neutrophils

The 24 h biofilms of *S. aureus* MR23 formed in glass-bottomed dishes as described above (See “Fluorescence staining of biofilms for CLSM”) were washed twice with 1 mL PBS. They were then incubated at 37 °C for 30 min in 5% CO_2_ with mouse neutrophils (4 × 10^6^ cells/mL) and suspended in 0.5 mL RPMI 1640 medium containing 10% (v/v) heat-inactivated fetal bovine serum (FBS; Nichirei Bioscience. Inc., Tokyo, Japan) and 1% (v/v) heat-inactivated mouse serum. After the removal of non-bound cells and media, the biofilms and neutrophils were fixed with 4% PFA at 37 °C for 30 min and washed twice with PBS. The specimens were incubated with 0.5 mL anti-Ly-6G-Alexa 647 (diluted 1:500 in PBS containing 2% (v/v) heat-inactivated FBS and 0.05% NaN_3_) at 4 °C overnight in the dark. After washing three times with PBS, the specimens were further stained with FM1-43 in PBS at 25 °C for at least 30 min or at 4 °C overnight in the dark. Finally, the biofilms and neutrophils were soaked in the indicated solutions and observed by CLSM, as described below.

### CLSM imaging

Three-dimensional biofilm structures were observed using an LSM880 confocal laser scanning microscope (Carl Zeiss) with alpha Plan-Apochromat ×100/1.46 Oil DIC M27 Elyra, Plan-Apochromat ×63/1.4 Oil DIC M27, Plan-Apochromat ×40/1.3 Oil DIC UV-IR M27, C-Apochromat ×40/1.2 W Korr FCS M27, Plan-Apochromat ×20/0.8 M27e, and Plan-Apochromat ×10/0.45 M27 objective lenses. Image acquisition was conducted at 405 (for DAPI), 488 (for ThT, FM1-43, and Alexa 488), 561 (for Alexa 594), and 633 nm (for Alexa 647). High-resolution images of biofilms were acquired on the LSM880 microscope with an Airyscan super-resolution unit (Carl Zeiss). Images were captured using a ×100 objective lens with digital zoom ×2, a ×63 objective lens with digital zoom from ×1.8 to ×5, ×40 objective lenses with digital zoom from ×1 to ×3, and a ×20 objective lens with digital zoom from ×1 to ×2, and z-sections were collected at intervals of 0.185 to 2 μm.

### Live-cell imaging for biofilm development

*S. epidermidis* SE21 was cultured in BHI (2 mL) at 37 °C overnight. The culture was diluted to 1:100 in BHIG, and the suspension was poured into a 35-mm glass-bottomed dish (Matsunami Glass). After preincubation at 37 °C for 4 h, the medium and planktonic cells were removed, and the attached cells were washed once with BHIG. Subsequently, BHIG (2 mL) containing 30.0% (w/v) iodixanol and 25 μM ThT was added to the dish. Live-cell imaging was performed on the LSM880 confocal scanning microscope with a Plan-Apochromat ×40/1.3 Oil DIC UV-IR M27 objective lens and an autofocus module. During imaging, the temperature of the biofilm culture dish was maintained at 37 °C using a large cage incubator and a stage top incubator. The 3D images were captured every 30 min, and Z-stacks were taken at 1.216-μm intervals. As a control, live cell imaging was performed using BHIG supplemented with 25 μM ThT, but without iodixanol. In addition, live cell imaging was performed using the LSM880 in reflection mode. In this case, no fluorescence dye was added to BHIG or BHIG containing 30.0% (w/v) iodixanol, and images were acquired at 633 nm.

Living biofilms of *S. aureus* and *C. albicans* were imaged using the THUNDER DMi8 microscope as described in Supplementary Note 1.

### Image processing

Three-dimensional structures were reconstructed using the microscope software ZEN 3.0 SR black (64 bit) for ZEISS microscopy (Carl Zeiss) and Imaris (Bitplane, Belfast, United Kingdom). For the rendering of three-dimensional images, the maximum projection mode was used for the comparison of treatments (Fig. 1 and Supplementary Fig. 2). The dynamic ranges of images were adjusted using the Min/Max function, which can help to automate and optimize the display range for each image. In the other cases, the transparent mode was used to show stereoscopic images, in which a three-dimensional image with a transparent effect was calculated.

Live-cell images taken on the Leica THUNDER DMi8 microscope were processed using the THUNDER Imaging System (Leica Microsystems, Wetzlar, Germany) in SVCC mode.

### ASEM imaging

ASEM images were recorded using the ClairScope ASEM system (JASM-6200, JEOL, Ltd, Tokyo, Japan)^33,49^ as described in Supplementary Note 1.

### Statistics and reproducibility

Statistical analysis was performed using GraphPad Prism Ver. 9 for Windows OS (GraphPad Software, Inc., San Diego, CA, USA). Student *t* test or one-way analysis of variance with Dunnett’s post hoc test were used to determine whether groups exhibited statistically significant differences in the effects of immersion solutions compared with non-treated control samples. All experiments were performed at least three times. The sample sizes are indicated in the figure legends and Supplementary Data 1. For all analyses, *P* < 0.05 was considered statistically significant.

### Reporting summary

Further information on research design is available in the Nature Portfolio Reporting Summary linked to this article.

## Supplementary information

Supplementary Information

Description of Additional Supplementary Files

Supplementary Movie 1

Supplementary Movie 2

Supplementary Movie 3

Supplementary Movie 4

Supplementary Movie 5

Supplementary Movie 6

Supplementary Movie 7

Supplementary Movie 8

Supplementary Movie 9

Supplementary Movie 10

Supplementary Movie 11

Supplementary Movie 12

Supplementary Movie 13

Supplementary Data 1

Reporting Summary

## Data Availability

All source data underlying the graphs presented in the figures are available in Supplementary Data 1 or available from the corresponding author on reasonable request.
