# Supplementary material for: Instantaneous Clearing of Biofilm (iCBiofilm): an optical approach to revisit bacterial and fungal biofilm imaging
Source: Commun Biol. 2023 Jan 23;6:38. doi: 10.1038/s42003-022-04396-4 (PMC9870912; doi:10.1038/s42003-022-04396-4)
Supplement: Supplementary file 1 — Supplementary Information [file 42003_2022_4396_MOESM1_ESM.pdf]

Supplementary Information

Instantaneous Clearing of Biofilm (iCBiofilm): an optical approach to revisit bacterial and fungal biofilm imaging

Shinya Sugimoto[1,2,*] and Yuki Kinjo[1,2]

[1]Department of Bacteriology, The Jikei University School of Medicine,
3-25-8 Nishi-Shimbashi, Minato-ku, Tokyo, 105-8461, Japan

[2]Jikei Center for Biofilm Science and Technology,
The Jikei University School of Medicine,
3-25-8 Nishi-Shimbashi, Minato-ku, Tokyo, 105-8461, Japan

*Correspondence: ssugimoto@jikei.ac.jp

This file contains Supplementary Note 1, Supplementary Tables 1 and 2, Supplementary Figures 1-12, and Supplementary References.

Supplementary Note 1

Mounting reagents

Immersion Solution M was purchased from CelExplorer Labs Co. (Hsinchu, Taiwan). SlowFade Diamond and ProLong Antifade Diamond were purchased from Thermo Fisher Scientific (Waltham, MA, USA). Super Clear Mount was purchased from FUJIFILM Wako Chemicals (Tokyo, Japan). Trolox was purchased from Cayman Chemical Company (Ann Arbor, MI, USA).

Clearing of fixed biofilms with CUBIC

CUBIC Trial Kit (FUJIFILM Wako Chemicals) was used to conduct biofilm clearing, according to the manufacturer instructions. The paraformaldehyde (PFA)-fixed biofilm of MR23 was incubated in 50% (v/v) Sca*l*eCUBIC Solution-1 at 25 °C for 6 h and 100% (v/v) Sca*l*eCUBIC Solution-1 at 37 °C for 24 h. After washing with PBS (-) at 37 °C for 6 h, the biofilm was further incubated in 50% (v/v) Sca*l*eCUBIC Solution-2 at 25 °C for 24 h and 100% (v/v) Sca*l*eCUBIC Solution-2 supplemented with 25 μM ThT at 25 °C for 24 h. Finally, the biofilm was soaked in mixture of Mounting Solution-1 and Mounting Solution-2 (2 : 8) and observed by CLSM.

Clearing of fixed biofilms with Sca*l*eS

SCALEVIEW-S (FUJIFILM Wako Chemicals) was used to conduct biofilm clearing, according to the manufacturer instructions. The PFA-fixed biofilm of MR23 was incubated in SCALEVIEW-S0 at 37 °C for 30 min, SCALEVIEW-S1 at 37 °C for 30 min, SCALEVIEW-S2 at 37 °C for 30 min, and SCALEVIEW-S3 at 37 °C for 30 min. After treatment with deScale Solution at 4 °C for 3 h, the biofilm was further incubated in SCALEVIEW-S4 at 37 °C for 16 h and SCALEVIEW-S4 supplemented with 25 μM ThT at 37 °C for 1 h. Finally, the biofilm was soaked in SCALEVIEW-SMt and observed by CLSM.

Atmospheric scanning electron micoscopy (ASEM)

Staphylococcus aureus MR23 and *Staphylococcus epidermidis* SE21 were grown in brain heart infusion (BHI) (Becton Dickinson, Franklin Lakes, NJ, USA) at 37 °C overnight with shaking at 150 rpm. The overnight cultures were diluted 1,000-fold into BHI and BHI supplemented with 30% (w/v) iodixanol (BHI+). These bacterial cells were

further incubated at 37 °C with shaking at 150 rpm. When optical density of the cultures in BHI reached 1.1–1.3, bacterial cells were harvested by centrifugation (20,000 × *g*, 10 min, 4 °C). For collection of bacterial cells grown in BHI+, bacterial cultures were diluted 10-fold in phosphate-buffered saline (PBS) to reduce the concentration of iodixanol before centrifugation, since high concentration of iodixanol prevented sedimentation of bacterial cells.

Candida albicans SC5314 colonies grown on an yeast-peptone-dextrose (YPD) plate at 30 °C for 3 days were suspended in Roswell Park Memorial Institute 1640 (RPMI) (Gibco; Thermo Fisher Scientific, Waltham, MA, USA) medium buffered with MOPS (pH 7.0) (RPMI-MOPS) to approximately 10^6–10^7 CFU/mL cells. The cells were grown in RPMI and RPMI supplemented with 30% (w/v) iodixanol (RPMI+) at 37 °C for 24 h with shaking at 150 rpm. Then, *C. albicans* cells were harvested by centrifugation (20,000 × *g*, 10 min, 4 °C). For collection of cells grown in RPMI+, the culture was diluted 10-fold in PBS before centrifugation.

Harvested bacterial and fugal cells were washed once with PBS and fixed with 1% glutaraldehyde (GA) and 4% PFA in PBS for 30 min at 22–25 °C. After centrifugation (10,000 × *g*, 10 min, 4 °C), fixed cells were washed once with distilled and deionized water (DDW) and suspended in DDW. Then, these cells were placed on ASEM membrane dishes (35 mm in diameter, JEOL, Tokyo, Japan). After incubation for 60 min at 22–25 °C, the planktonic and weakly attached cells were removed by washing with DDW. The remainining cells were stained with positively charged nanogold (PCG) and phosphotungstic acid (PTA). The DDW-washed specimens were incubated with a 6 μM PCG solution (Nanoprobes, Yaphank, NY, USA) at for 20 min at 22–25 °C. The specimens were then washed with DDW and the size of the gold nanoparticles was increased by gold enhancement using the GoldEnhance kit (Nanoprobes) for 10 min at 22–25 °C, followed by an additional washing in DDW. The resultant specimens were then incubated in 2% (w/v) PTA (TAAB Laboratories Equipment, Aldermaston, Berks, England) at 4 °C overnight. After three times washig with DDW, specimens were soaked in 1% (w/v) ascorbic acid in DDW and observed using ASEM.

ASEM images were recorded using the ClairScope ASEM system (JASM-6200, JEOL, Ltd, Tokyo, Japan)[1, 2]. The acceleration voltage of the SEM was 30 kV, and backscattered electrons (BSE) from the specimens were recorded by a BSE imaging (BEI) detector to visualise the sample.

S. aureus growth analysis

S. aureus MR23 was grown in 2 mL of BHI medium at 37 °C for 16–20 h. The culture was diluted 1:1000 in fresh BHI (2 mL) and supplemented with 28.1% (w/w) iohexol or 30.0% (w/v) iodixanol, then cultured at 37 °C for 24 h. Aliquots (5 μL) of the 10-fold serial dilutions of the cultures were spotted on LB agar plates and incubated at 37 °C for 24 h. After incubation, digital photos were taken.

Single-cell analysis for cell division of bacteria and elongation speed of fugal hyphae

S. aureus MR23 and *S. epidermidis* SE21 were grown in BHI at 37 °C overnight with shaking at 150 rpm. The overnight cultures were dilutted 100-fold in BHI and the small aliquots (2 μL) were spotted on BHI agar plates and BHI agar plates supplemented with 30% (w/v) iodixanol (BHI+).

C. albicans SC5314 colonies grown on an YPD plate at 30 °C for 3 days were suspended in RPMI-MOPS to approximately 10^4–10^5 CFU/mL cells. A small aliquot (2 μL) of the cell suspension was spotted on RPMI agar plates or RPMI agar plates supplemented with 30% (w/v) iodixanol (RPMI+).

Small blocks of the agar plates inoculated with the bacteria and fungus were invertedly placed on glass-bottommed dished (Matsunami Glass, Osaka, Japan). These bacterial and fungal cells were incubated at 37 °C and continuously imaged every minute for more than 6 h using a phase contrast DMi8 microscope (Leica Microsystems, Wetzlar, Germany).

Live-cell imaging for biofilm formation of *S. aureus* and *C. albicans*

Living biofilms of *S. aureus* and *C. albicans* could not be analyzed using the LSM880 microscope in the basic research facility of the Jikei University School of Medicine because these pathogens (biosafety level 2) were not permitted in the facility (P1). Live cell imaging of these biofilms was performed using a THUNDER DMi8 microscope (Leica Microsystems), which was recently established in our laboratory (P2).

Live cell imaging of *S. aureus* biofilm development was performed as follows: *S. aureus* MR23 was grown in BHI (2 mL) at 37 °C overnight. The culture was diluted to 1:100 in BHIG supplemented with 1 μM MitoTracker Deep Red FM (Thermo Fisher Scientific), and the suspension was added to a 35-mm glass-bottomed dish

(Matsunami Glass). After incubation at 30 °C for 2 h, the medium and planktonic cells were removed, and the attached cells were washed once with BHIG to remove remaining non-adherent cells. Subsequently, BHIG (4 mL) containing 15.0% (w/v) iodixanol and 1 μM MitoTracker Deep Red was added to the dish. Live cell imaging was performed on a THUNDER DMi8 microscope using a ×63 oil immersion objective lens (NA 1.40) in SVCC mode. Three-dimensional images were captured every 10 min at 30 °C for 24 h. Z-stacks were taken at 1-μm intervals.

Live cell imaging of the development of *C. albicans* biofilms was performed as follows: *C. albicans* SC5314 cultured on YPD plates at 25 °C for 3 days was suspended in RPMI-MOPS (2 mL). The suspension, containing approximately 10^7 CFU/mL, was added to a 35 mm glass-bottomed dish (Matsunami Glass). After preincubation at 30 °C for 30 min, the medium and planktonic cells were removed, and the adherent cells were washed once with RPMI-MOPS to remove remaining non-adherent cells. RPMI-MOPS (5 mL) containing 24.0% (w/v) iodixanol and 1 μM MitoTracker Deep Red was added to the dish. Imaging was performed on the THUNDER DMi8 microscope using a ×20 objective (NA 0.8) in THUNDER SVCC mode. Three-dimensional images were captured every 15 min at 30 °C for 24 h. Z-stacks were taken at 2 μm intervals.

Live-cell imaging for response of biofilms to antimicrobials

Live cell imaging of *S. aureus* biofilm response to antimicrobials was performed as follows: Mature 24 h biofilms of MR23 formed in 35 mm glass-bottomed dishes (Matsunami Glass) were washed three times with 1 mL DDW and stained using the FilmTracer LIVE/DEAD Biofilm Viability Kit (Thermo Fisher Scientific) according to manufacturer instructions. After incubation at 25 °C for 30 min, the fluorescent dyes (SYTO 9 and PI) were replaced with fresh dyes (1 mL) containing SYTO 9, PI, 15.0% (w/v) iodixanol, 1 μM Trolox, and PBS. Trolox was added to minimize fluorescence decay during observation. A total of 200 μM vancomycin (20× MIC) and 50 μM nisin A (20× MIC), were added separately. Immediately after adding these solutions, live cell imaging was performed using the THUNDER DMi8 microscope with a ×20 objective (NA 0.8) in SVCC mode. Three-dimensional images were captured every 1 min at 30 °C for 1 h. Z-stacks were taken at 2 μm intervals.

Supplementary Table 1. Microorganisms and plasmids used in this study.

Strains and plasmids	Description[a]	Source or reference
S. aureus		
RN4220	NCTC8325-4 derivative, restriction deficient mutant	3
MR4	A clinical isolate of MRSA from a patient in the Jikei hospital; an eDNA and protein-dependent biofilm producer	4
MR10	A clinical isolate of MRSA from a patient in the Jikei hospital; a PIA-dependent biofilm producer	4, 5
MR23	A clinical isolate of MRSA from a patient in the Jikei hospital; a protein-dependent biofilm producer	5
MR23 Δ*spa* Δ*sbi*	*spa* and *sbi* were deleted from MR23	2
MR23 Δ*spa* Δ*sbi* Δ*eap*	*spa*, *sbi*, and *eap* were deleted from MR23	2
MR23 Δ*spa* Δ*sbi eap-mS1*	The gene encoding mScarlet-1 was inserted at the downstream of *eap* in the genome of MR23 Δ*spa* Δ*sbi;* expressing Eap-mScarlet-1 transcriptional fusion	This study
MR23 Δ*spa* Δ*sbi sasG-mNG*	The gene encoding mNeonGreen was inserted at the downstream of *sasG* in the genome of MR23 Δ*spa* Δ*sbi;* expressing SasG-mNeonGreen transcriptional fusion	This study
MR23 Δ*spa* Δ*sbi eap*-mS1 *sasG-mNG*	The gene encoding mNeonGreen was inserted at the downstream of *sasG* in the genome of MR23 Δ*spa* Δ*sbi eap-mS1;* expressing Eap-mScarlet-1 and SasG-mNeonGreen transcriptional fusions	This study
S. epidermidis		
SE21	A clinical isolate of MRSA from a patient in the Jikei hospital; a PIA-dependent biofilm producer	4
E. coli		
DH5α	*fhuA2* Δ(*argF-lacZ*)*U169 phoA glnV44 φ80* Δ(*lacZ*)*M15 gyrA96 recA1 relA1 endA1 thi-1 hsdR17*	Toyobo, Osaka, Japan
BW25113	Δ(*araD-araB*)*567* Δ(*rhaD-rhaB*)*568 ΔlacZ4787* (::rrnB-3) *hsdR514 rph-1* The wild-type K-12 strain of Keio collection	6
C. albicans		
SC5314	Type strain	
Plasmid		
pEX-K4J2	Cloning vector, Km^R	Eurofins
pEX-eap-mS1	The DNA containing the 3'-end 500-bp fragment of MR23 *eap*, ribosome-binding site, the mScarlet-1 gene, and the 500-bp fragment of the downstream region of MR23 *eap*	This study
pEX-sasG-mNG	The DNA containing the 3'-end 500-bp fragment of MR23 *sasG*, ribosome-binding site, the mNeonGreen gene, and the 500-bp fragment of the downstream region of MR23 *sasG*	This study

pKOR1	*E. coli-S. aureus* shuttle vector plasmid for knockout of genes by allelic exchange, Amp^R, Cm^R	7
pKOR1-eap-mS1	pKOR1-derivative plasmid for knock-in of the gene encoding mScarlet-1 with the ribosome-binding site at the downstream of *eap* in the genome of MR23, Amp^R, Cm^R	This study
pKOR1-sasG-mNG	pKOR1-derivative plasmid for knock-in of the gene encoding mNeonGreen with the ribosome-binding site at the downstream of *sasG* in the genome of MR23, Amp^R, Cm^R	This study

[a]Amp^R, ampicillin-resistance; Cm^R, chloramphenicol-resistance.

Supplementary Table 2. Oligonucleotide primers used in this study.

Primers	Sequence (5' to 3')	Description
eap-mScarlet-pKOR1-F	GGGGACAAGTTTGTACAAAAAAGCAGGCTGTATCGGGGAACGTGAATTAAAATATGCAAAAAAAGC	Forward primer, for cloning of the mScarlet-1 gene in pKOR1
eap-mScarlet-pKOR1-R	GGGGACCACTTTGTACAAGAAAGCTGGGTAATTAGTTTTATACATCTCTCCTAACAAAATCAAAAAATCGGA	Reverse primer, for cloning of the mScarlet-1 gene in pKOR1
sasG-mNeon-pKOR1-F	GGGGACAAGTTTGTACAAAAAAGCAGGCTAACGTGAGTTTAATCCAAAATTACAACCTGGTGAAG	Forward primer, for cloning of the mNeonGreen gene in pKOR1
sasG-mNeon-pKOR1-R	GGGGACCACTTTGTACAAGAAAGCTGGGTATTCAACAGTAAACCAAATTATTTGTAACGAAAACAGCAC	Reverse primer, for cloning of the mNeonGreen gene in pKOR1

Supplementary Figures

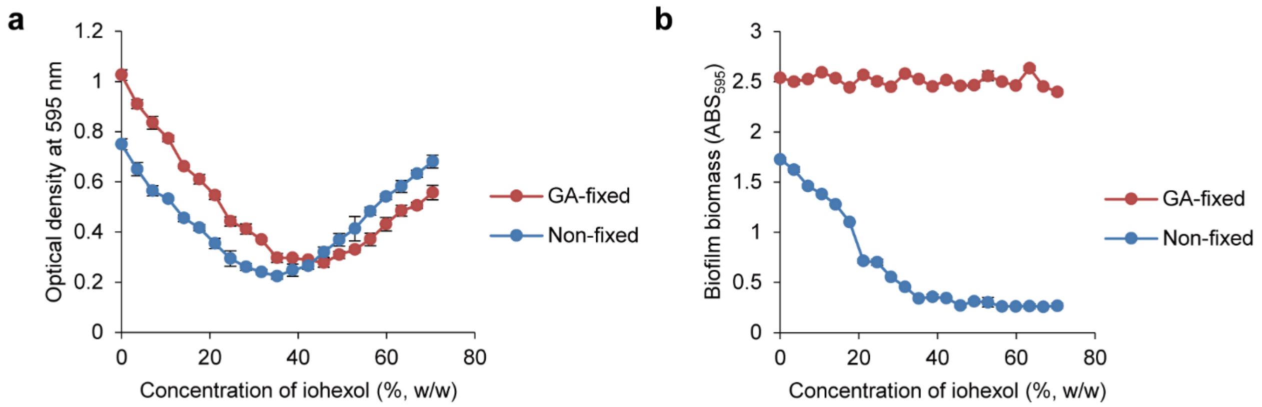

Supplementary Fig. 1. Effects of iohexol on absorbance and stability of *S. aureus* biofilms.
(**a**) Optical density vs iohexol concentration for glutaraldehyde (GA)-fixed and non-fixed *S. aureus* MR4 biofilms. (**b**) Effects of iohexol on stability of GA-fixed and non-fixed MR4 biofilms. Means and standard deviations (error bars) are shown (n = 4).

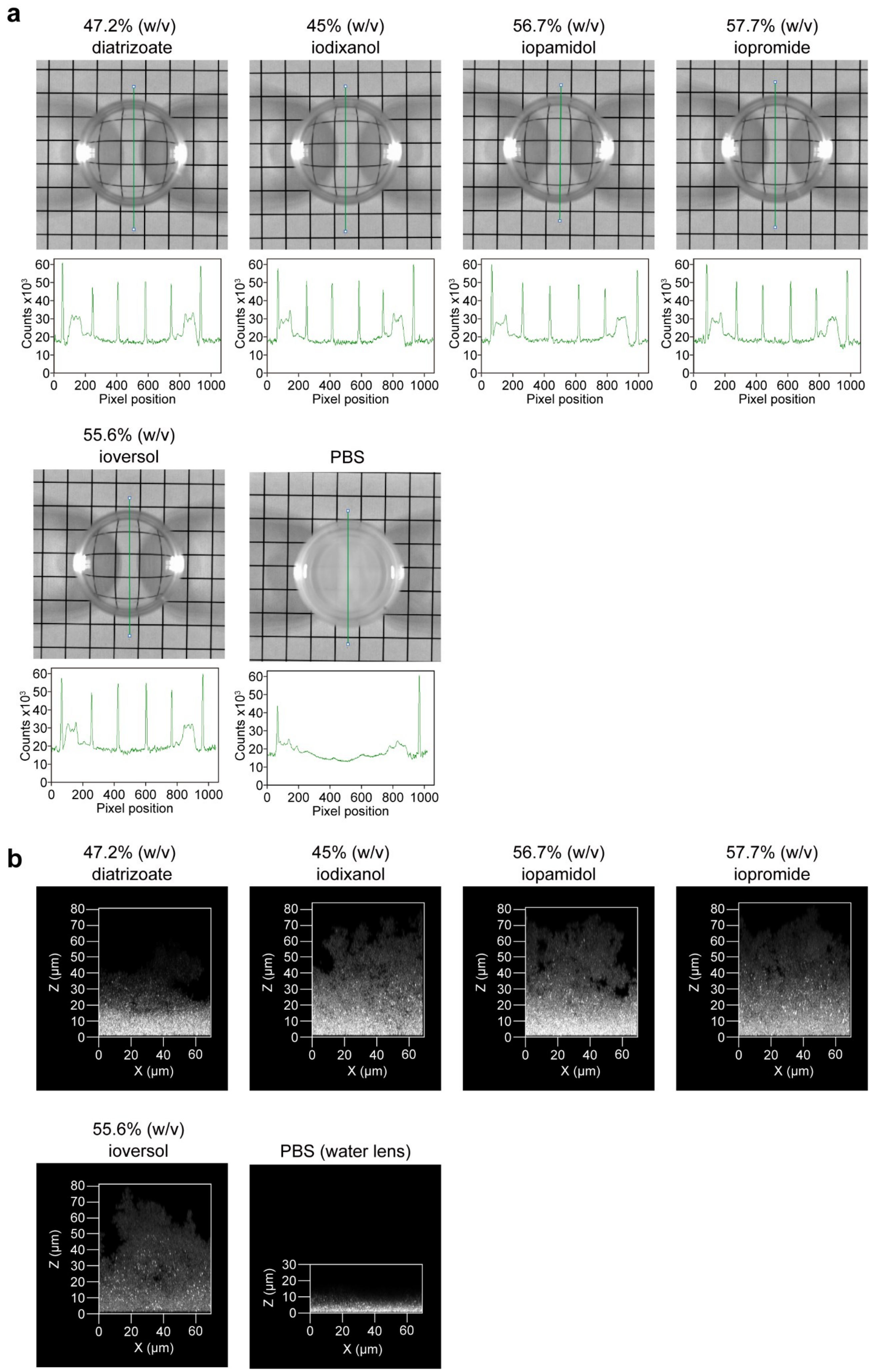
a
47.2% (w/v) diatrizoate
45% (w/v) iodixanol
56.7% (w/v) iopamidol
57.7% (w/v) iopromide
55.6% (w/v) ioversol
PBS
Counts x10³
Pixel position
b
47.2% (w/v) diatrizoate
45% (w/v) iodixanol
56.7% (w/v) iopamidol
57.7% (w/v) iopromide
55.6% (w/v) ioversol
PBS (water lens)
Z (µm)
X (µm)

Supplementary Fig. 2. Effects of various RI-matching media on clearing of biofilms.
(**a**) Photographs of MR23 biofilms on a grid pattern-printed paper as a background were obtained using ImageQuant for transmission. Plot profiles for the biofilms immersed in the indicated solutions are also shown in the lower panels. The green line in the upper image indicates the corresponding location of the plot profile in the lower panel, respectively. (**b**) Typical side-views of the MR23 biofilms fixed with 4% PFA, stained with FM1-43, and soaked in the indicated solutions. An LSM880 confocal laser scanning microscope with a ×40 objective lens and an Airyscan super-resolution unit was used to acquire z-stacks of the stained biofilms every 0.22 μm. X and Z represent width and thickness, respectively.

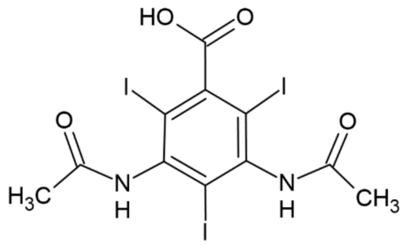

Diatrizoate

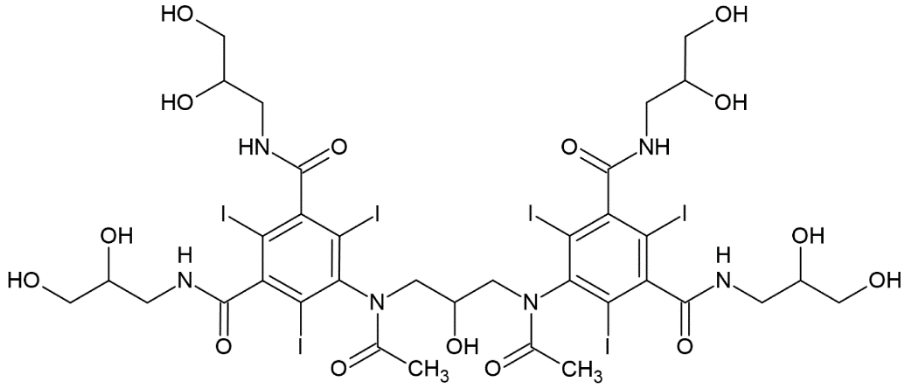

Iodixanol

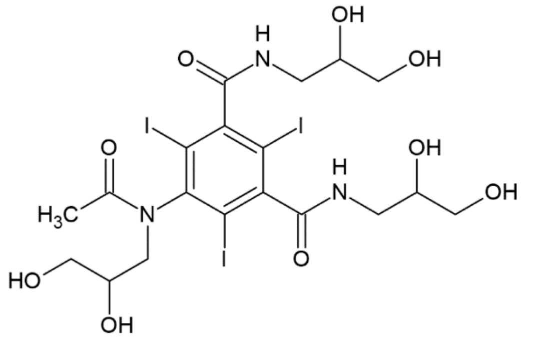

Iohexol

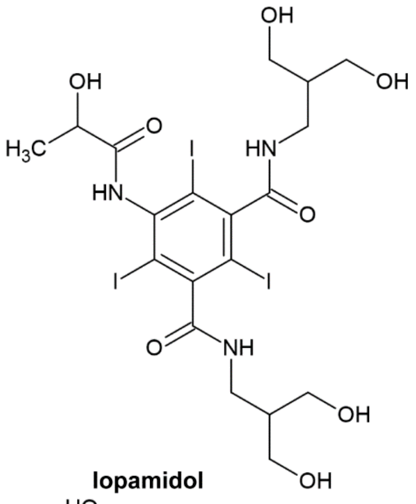

Iopamidol

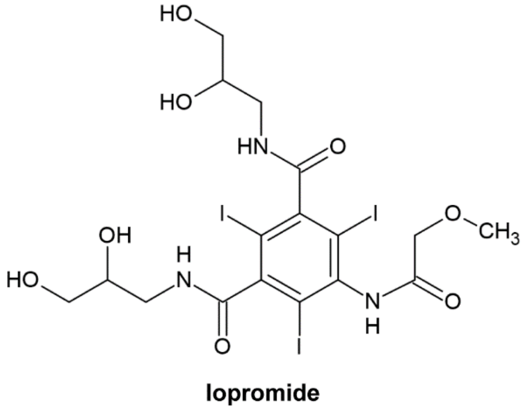

Iopromide

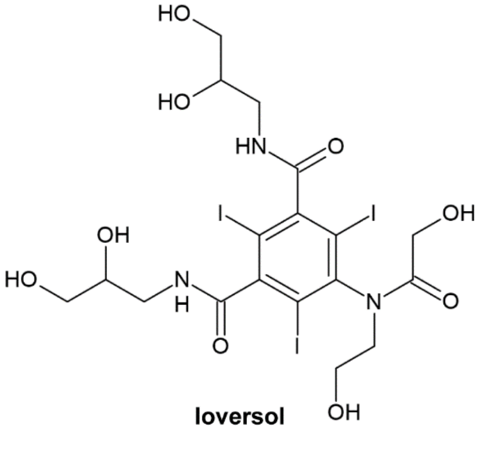

Ioversol

Supplementary Fig. 3. Chemical structures of RI-matching media used for biofilm clearing in this study.

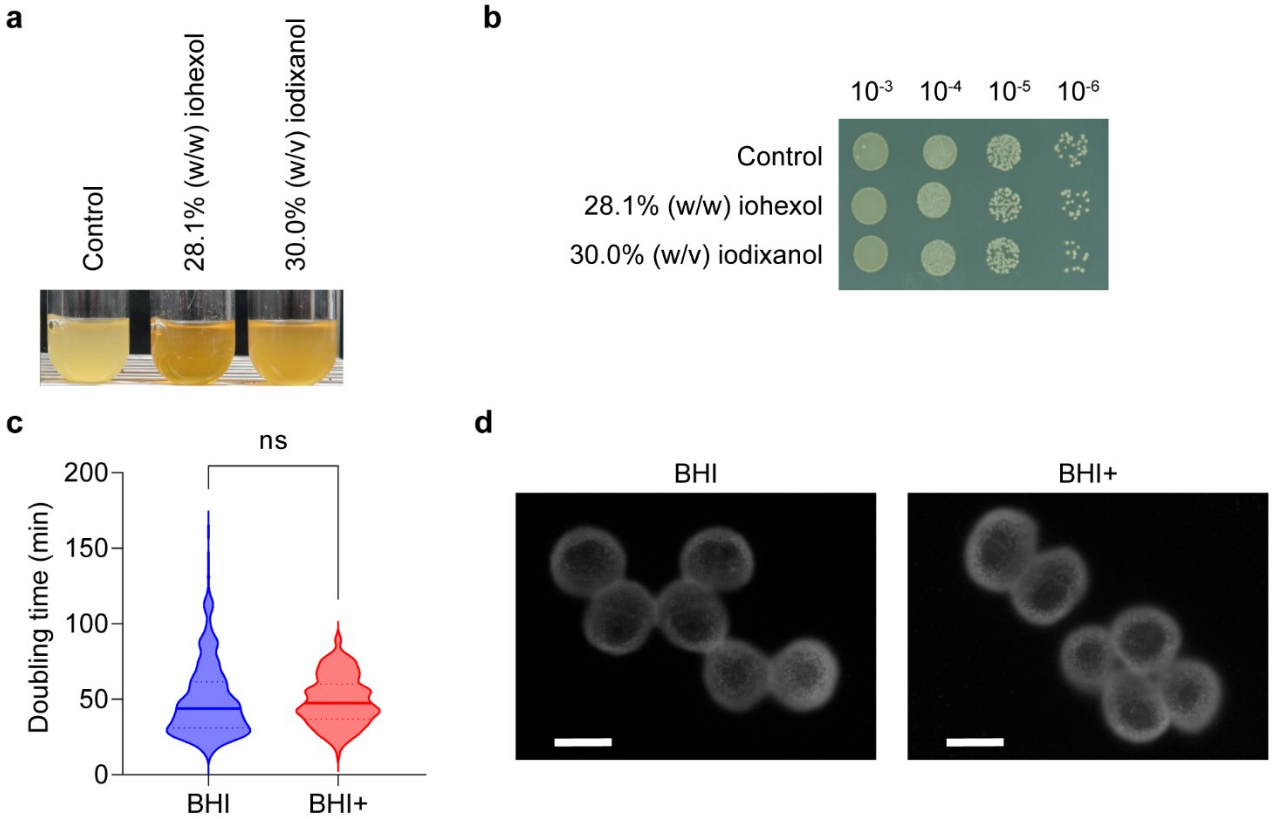

Supplementary Fig. 4. Effects of iohexol and iodixanol on the growth and biofilm structure of *S. aureus*.
(**a**) *S. aureus* MR23 grown in brain heart infusion medium (BHI, Control), BHI containing 28.1% (w/w) iohexol, or BHI containing 30.0% (w/v) iodixanol at 37 °C for 24 h. (**b**) Series of 10-fold dilutions of the bacterial cultures in **a** were spotted on LB agar plates and incubated at 37 °C for 24 h. (**c**) Violin plots of the doubling time of *S. aureus* MR23 cells measured by optical microscopy at the single-cell level. Cells were grown on BHI agar plates in the absence (BHI, n = 297) and presence of 30.0% (w/v) iodixanol (BHI+, n = 206) under the phase-contrast microscope. ns, not significant (unpaired two-tailed Student *t*-test). (**d**) ASEM images of MR23 cells grown in BHI liquid medium or BHI supplemented with 30.0% (w/v) iodixanol (BHI+). Typical views are shown. Scales, 500 nm.

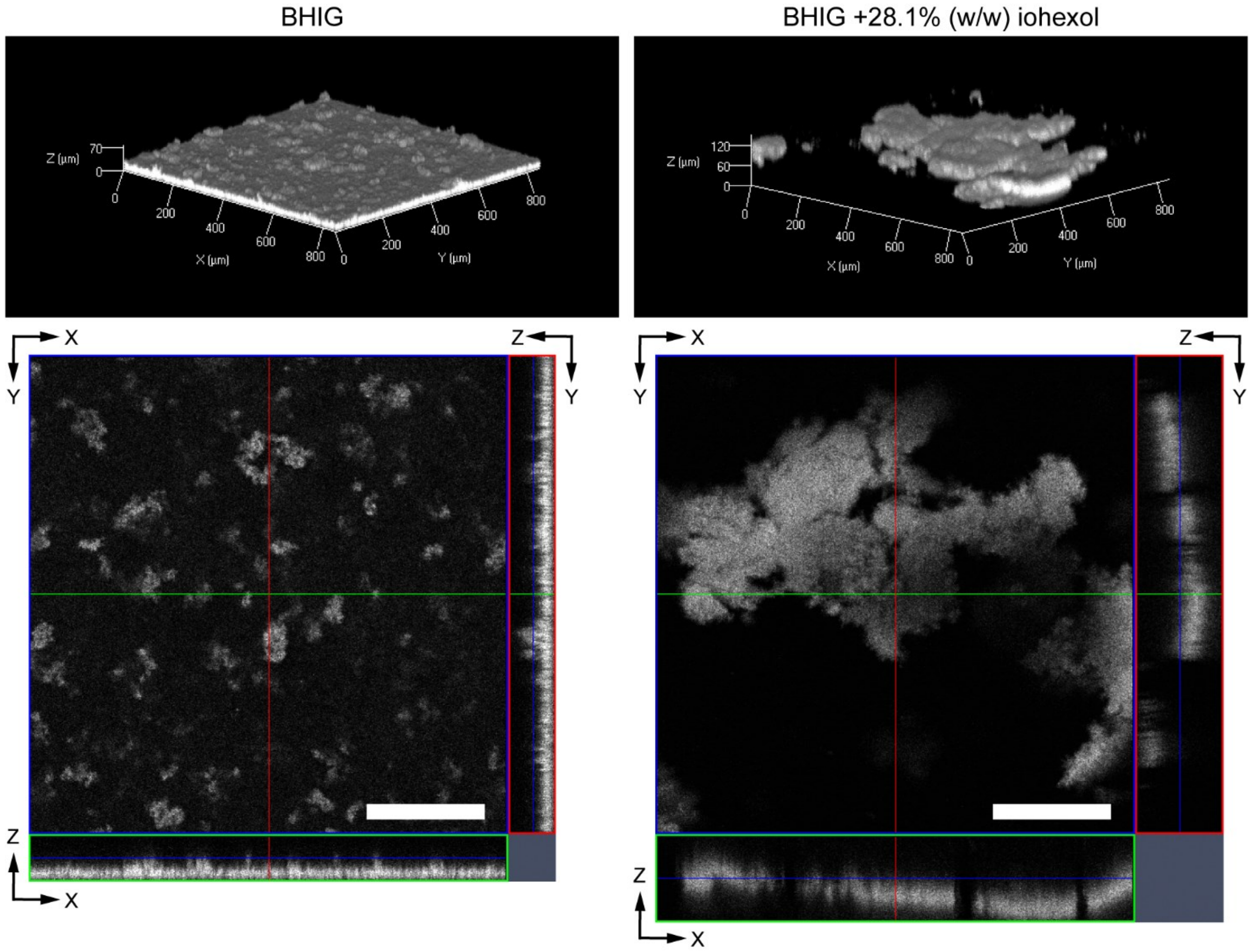

Supplementary Fig. 5. Effects of iohexol on the structure of the MR23 biofilm.
MR23 biofilms formed in BHIG or BHIG containing 28.1% (w/w) iohexol at 37 °C for 24 h, fixed with 1% glutaraldehyde at 25 °C for 30 min, and washed three times with PBS. After staining with FM1-43 at 25 °C for 30 min, the biofilms were soaked in 35.2% (w/w) iohexol and observed using an LSM880 microscope with a ×10 objective lens. Typical 3D and orthogonal images represent formation of weakly-attached and floating biofilms in the presence of 28.1% (w/w) iohexol. Scales, 200 µm.

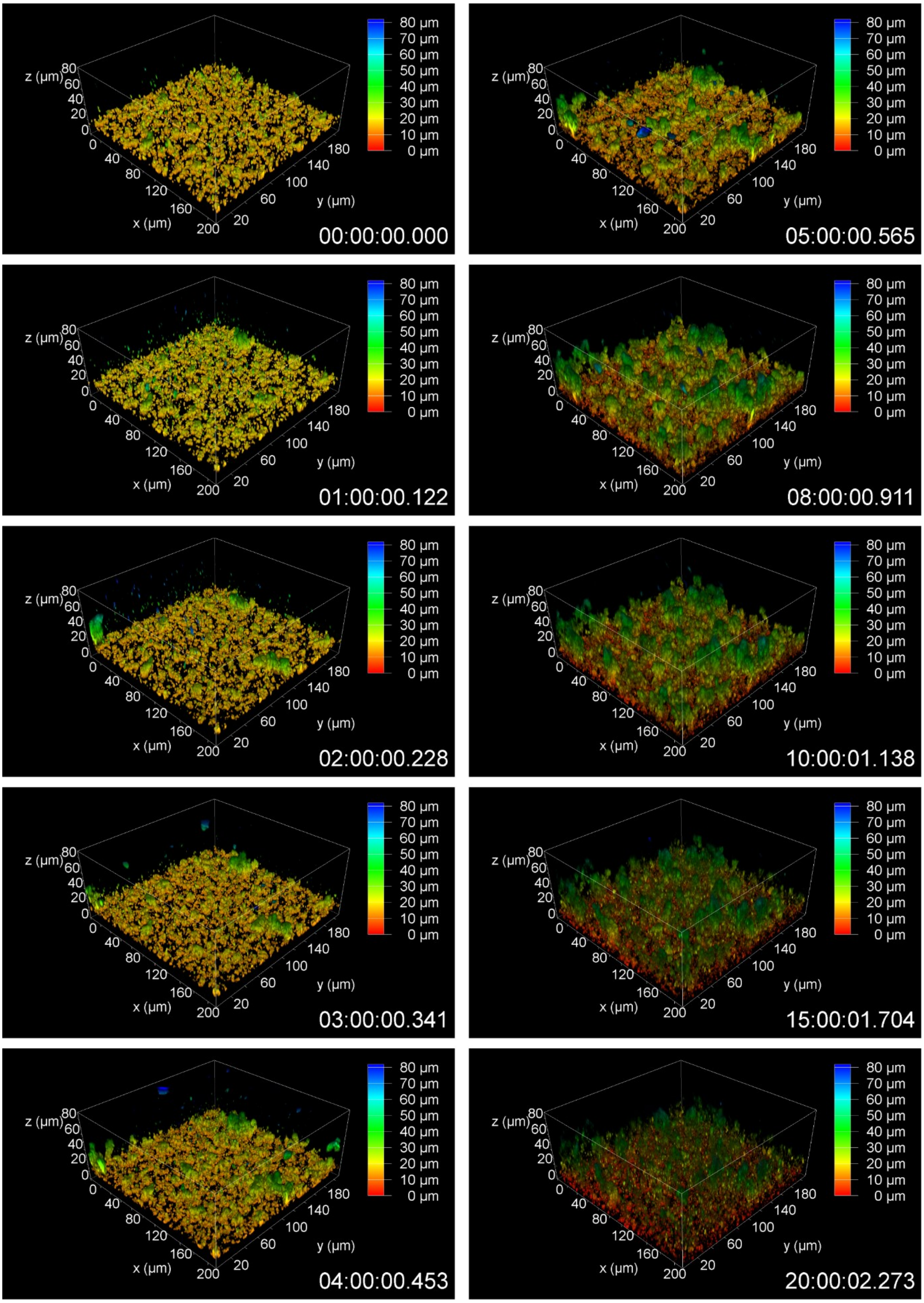

Supplementary Fig. 6. Live and dynamic imaging of *S. aureus* biofilm formation using iCBiofilm. Biofilm of *S. aureus* MR23 formed in BHIG containing 15.0% (w/v) iodixanol and 1 µM MitoTracker Deep Red. Imaging was performed every 15 min at 30 °C on a Leica Thunder DMi8 microscope using a ×63 oil immersion objective (NA 1.4) with Thunder SVCC (small volume computational clearing) mode; optical sections were acquired every 1 µm. 3D images at the indicated time points are shown in a heat map, with the color scale representing the thickness of the biofilm.

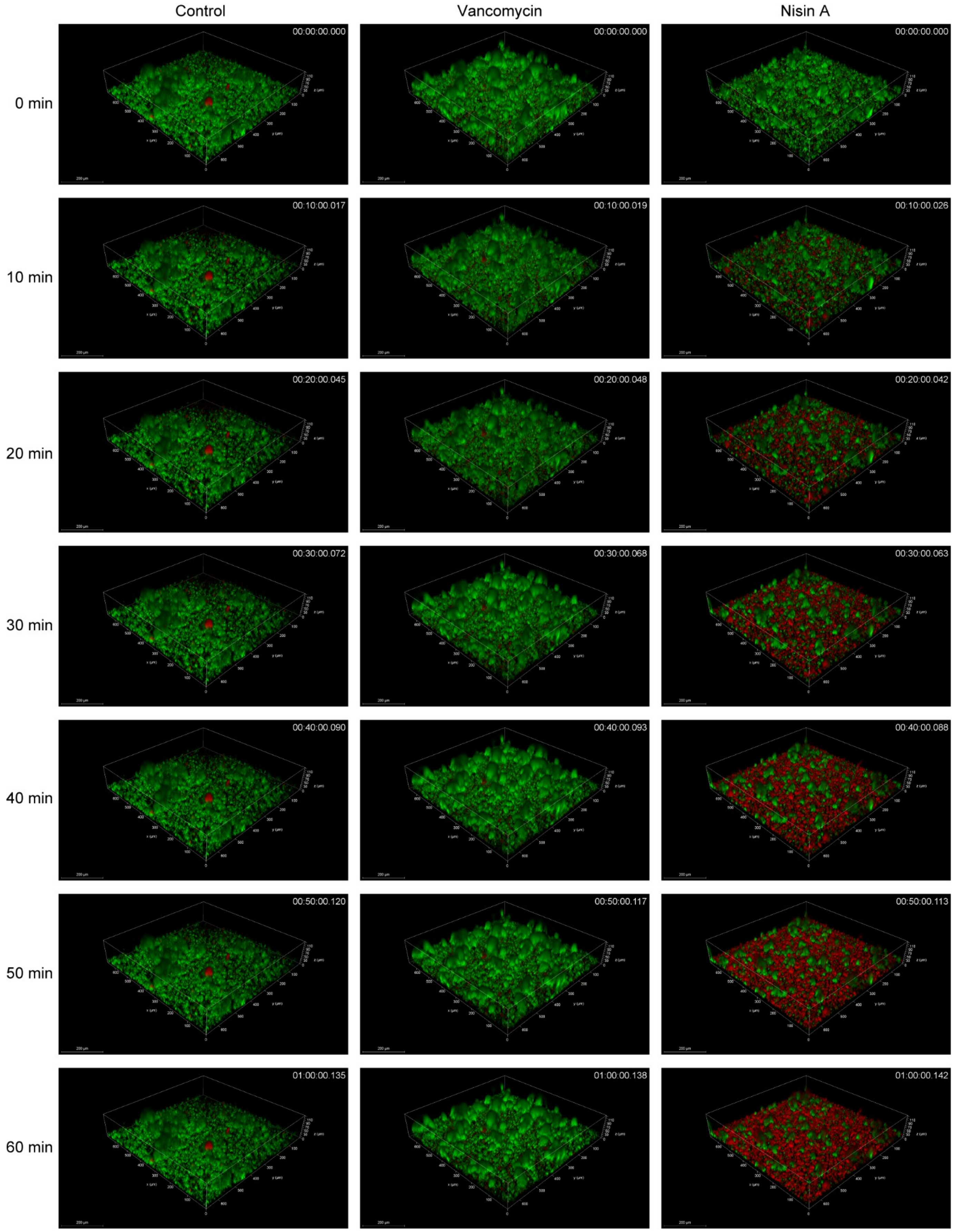

Supplementary Fig. 7. Imaging of biofilm response to antimicrobial agents using iCBiofilm.
Live cell clearing imaging for *S. aureus* MR23 biofilms in phosphate buffered saline containing 15.0% (w/v) iodixanol and the indicated antimicrobial agents in a glass-bottomed dish. Live cells were stained with SYTO9 (green), while dead or membrane-damaged cells were stained with PI (red). As a control, the biofilm was left untreated. 3D images at the indicated time points are shown.

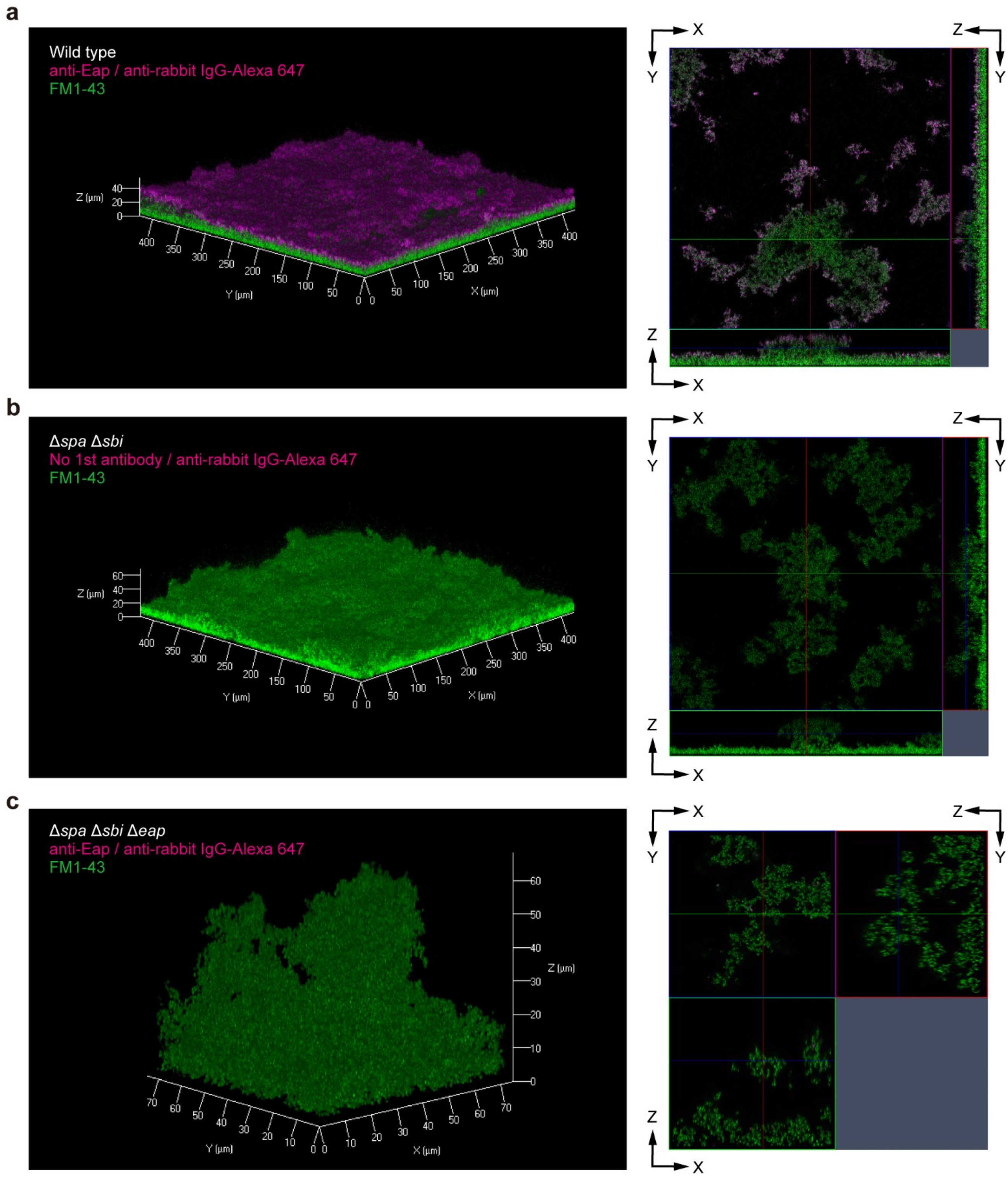

Supplementary Fig. 8. Indirect immunofluorescent microscopy for *S. aureus* biofilm.
Biofilms of *S. aureus* MR23 wild type (**a**), Δ*spa* Δ*sbi* (**b**), and Δ*spa* Δ*sbi* Δ*eap* (**c**) were labeled with anti-Eap rabbit antibody (**a** and **c**) or left untreated (**b**). The biofilms were further labeled with Alexa 647-conjugated goat anti-rabbit IgG and stained with FM1-43. Finally, the biofilms were soaked in 49.4% (w/v) ioversol solution and observed using an LSM880 microscope with ×20 (**a**, **b**) and ×63 (**c**) objective lenses and an Airyscan super-resolution unit. Typical 3D and orthogonal images are shown.

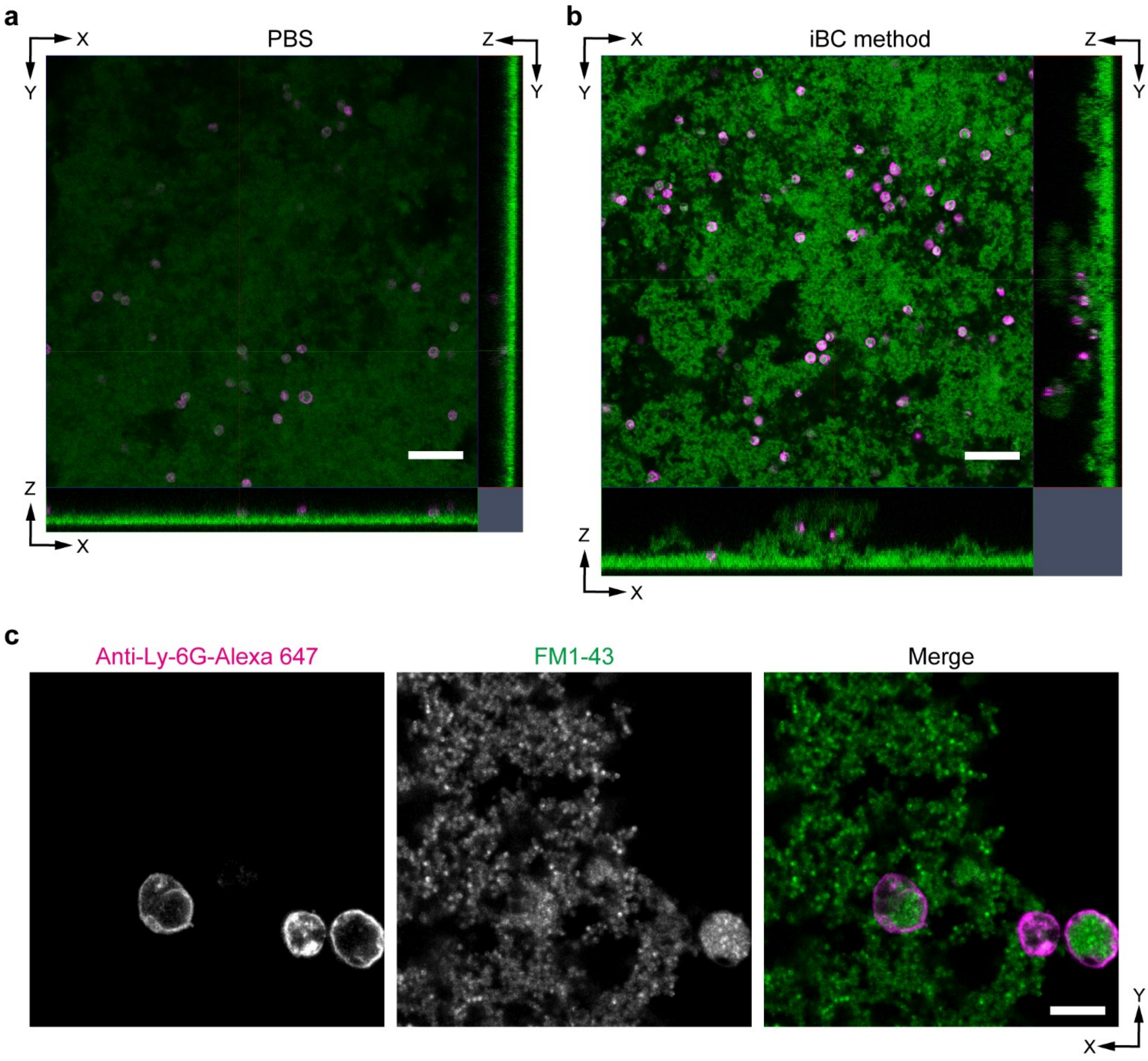

Supplementary Fig. 9. Interaction between *S. aureus* biofilms and mouse neutrophils.
(**a**-**c**) Biofilms of *S. aureus* MR23 and neutrophils were observed using an LSM880 microscope with a ×20 objective lens. The specimens were soaked in phosphate buffered saline (PBS) (**a**) or 37.1% (w/v) ioversol (**b**-**c**). The images acquired using the ×63 objective lens represent phagocytosis of *S. aureus* cell clusters in the biofilm by neutrophils (**c**). Green, *S. aureus* cells. Magenta, neutrophils. Scale bars are 50 μm in **a** and **b** and 10 μm in **c**.

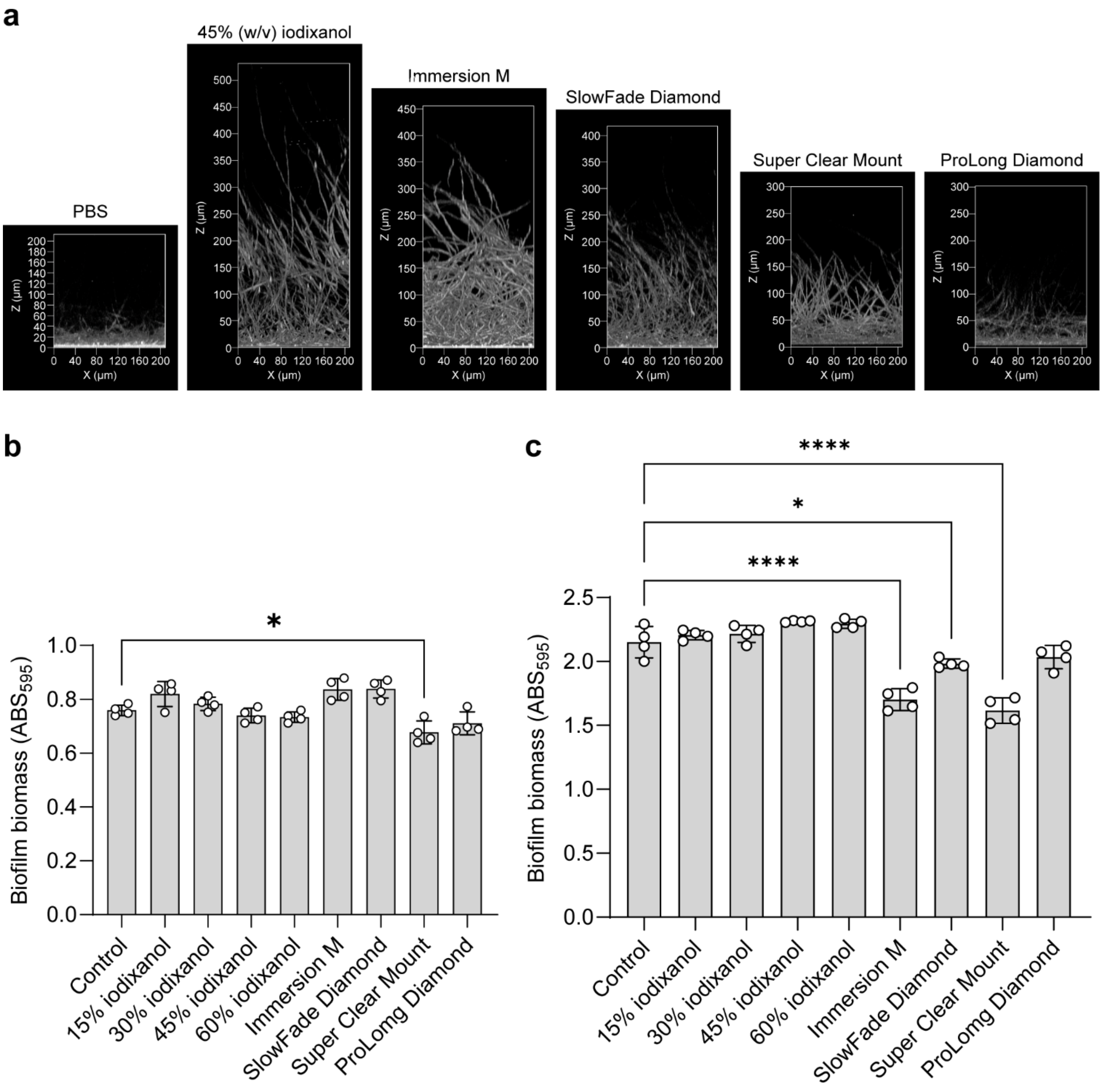

Supplementary Fig. 10. Comparison of immersion solutions for observation of biofilms.
(**a**) *C. albicans* SC5314 biofilms were grown in RPMI-MOPS (pH 7.0) at 37 °C for 48 h, stained with Con A-Alexa 594, and soaked in the indicated solutions. The biofilms were observed using an LSM880 microscope with a ×20 objective lens and an Airyscan super-resolution unit. Side views of the biofilms are shown. (**b**) Biomass of PFA-fixed *C. albicans* biofilms after treatment with various mounting reagents. (**c**) Biomass of PFA-fixed *S. aureus* MR4 biofilms after treatment with various mounting reagents. As a control, phosphate buffered saline was used. Means and standard deviations (error bars) are shown (n = 4). *P < 0.05; ****P < 0.0001 (one-way ANOVA followed by Dunnett's post hoc tests).

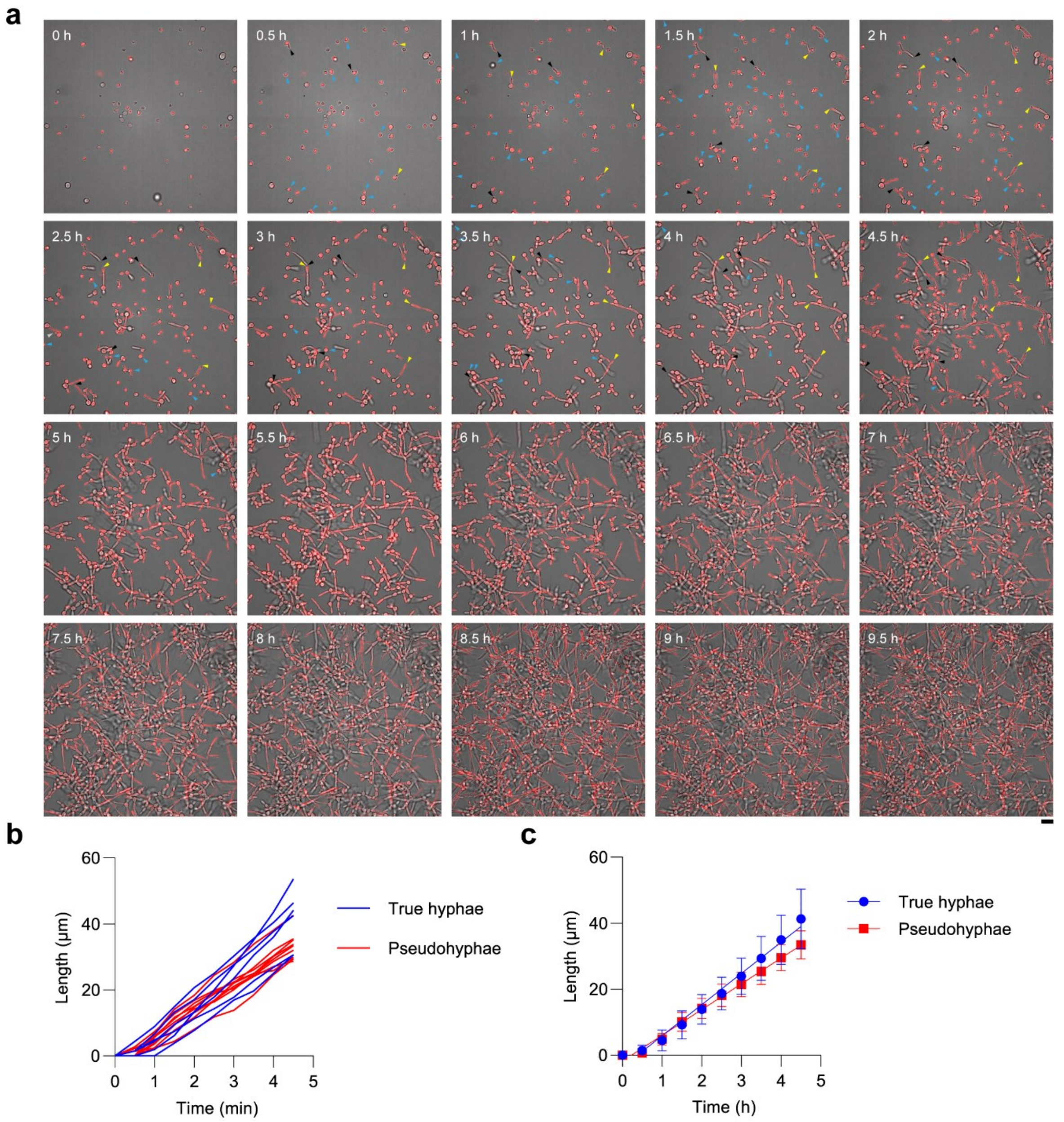

Supplementary Fig. 11. Single-cell analysis for growth of *C. albicans* cells in the biofilm.
(**a**) Growth and morphological change of glass surface-attached *C. albicans* SC5314 cells in the biofilm. Cells were cultured and imaged as described in Fig. 8. Merged images of MitoTracker Deep Red fluorescence and phase contrast at the indicated time points are shown. Black, yellow, and light blue arrowheads indicate true hyphae, peudohypahe, and newly surfce-attaching cells, respectively. (**b, c**) Elongation of true hyphae (n = 6) and pseudohyphae (n = 9) in the biofilm during the initial 4.5 h was analyzed at the single-cell level. Individual data (**b**) and Means ± standard deviations (**c**) are shown.

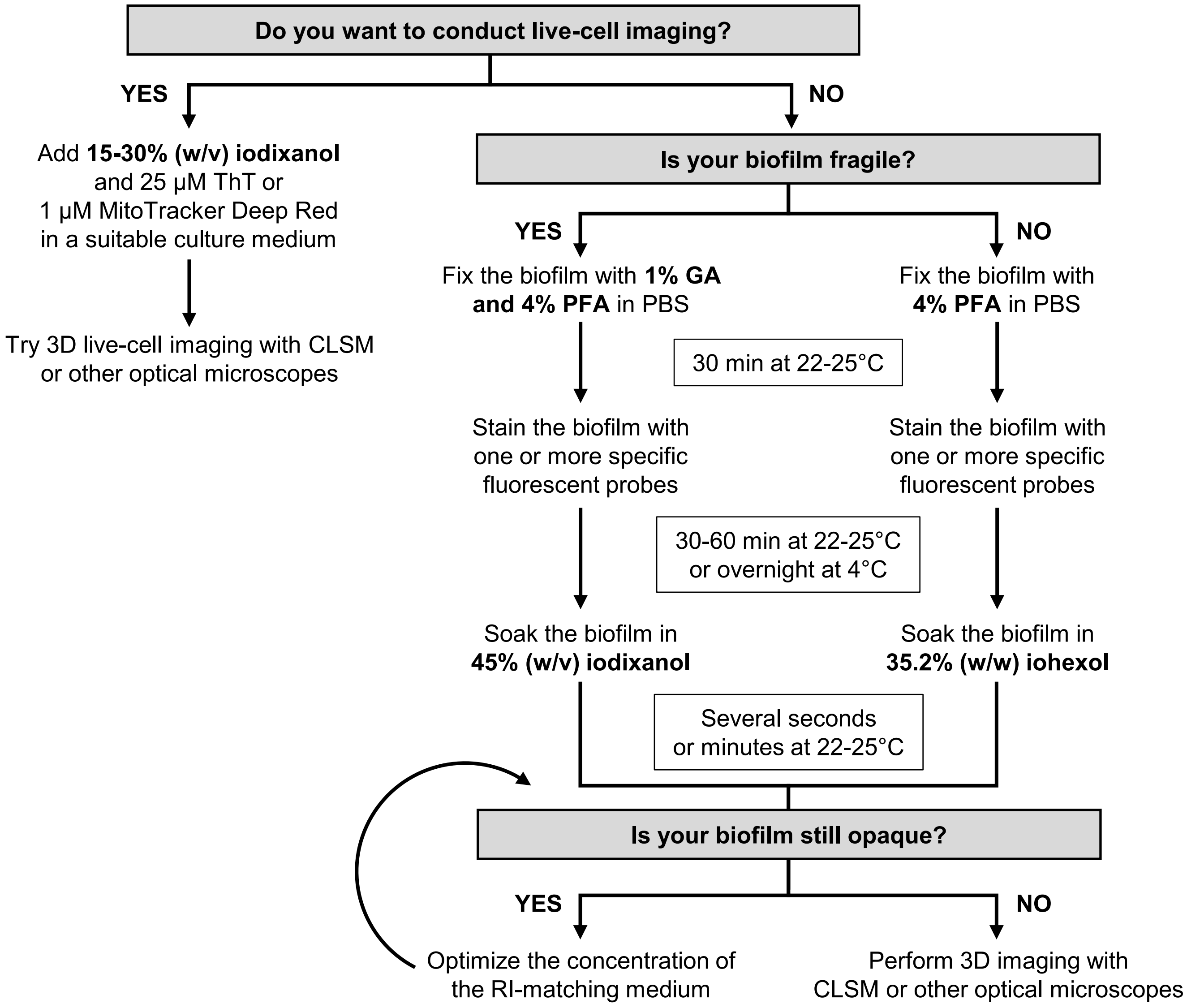

Supplementary Fig. 12. A flowchart for performing iCBiofilm and development of customized biofilm clearing.

Supplementary References

1. Nishiyama, H. et al. Atmospheric scanning electron microscope observes cells and tissues in open medium through silicon nitride film. *J. Struct. Biol.* **169**, 438-449 (2010).
2. Sugimoto, S. et al. Imaging of bacterial multicellular behaviour in biofilms in liquid by atmospheric scanning electron microscopy. *Sci. Rep.* **6**, 25889 (2016).
3. Kreiswirth, B. N. et al. The toxic shock syndrome exotoxin structural gene is not detectably transmitted by a prophage. *Nature* **305**, 709–712 (1983).
4. Sugimoto, S. et al. Broad impact of extracellular DNA on biofilm formation by clinically isolated Methicillin-resistant and -sensitive strains of *Staphylococcus aureus*. *Sci. Rep.* **8**, 2254 (2018).
5. Sugimoto, S. et al. *Staphylococcus epidermidis* Esp degrades specific proteins associated with *Staphylococcus aureus* biofilm formation and host-pathogen interaction. *J. Bacteriol.* **195**, 1645-1655 (2013).
6. Arita-Morioka, K., Yamanaka, K., Mizunoe, Y., Ogura, T. & Sugimoto, S. Novel strategy for biofilm inhibition by using small molecules targeting molecular chaperone DnaK. *Antimicrob. Agents Chemother.* **59**, 633-641 (2015).
7. Bae, T. & Schneewind, O. Allelic replacement in *Staphylococcus aureus* with inducible counter-selection. *Plasmid* **55**, 58-63 (2006).
