# Supplementary material for: Instantaneous Clearing of Biofilm (iCBiofilm): an optical approach to revisit bacterial and fungal biofilm imaging
Source: Commun Biol. 2023 Jan 23;6:38. doi: 10.1038/s42003-022-04396-4 (PMC9870912; doi:10.1038/s42003-022-04396-4)
Supplement: Supplementary file 2 — Description of Additional Supplementary Files [file 42003_2022_4396_MOESM2_ESM.pdf]

Description of Additional Supplementary Files

File name: Supplementary Data 1
Description: The source data behind the graphs in the paper.

File name: Supplementary Movie 1
Description: CLSM imaging of the MR23 biofilm stained with ThT and cleared with 35.2% (w/w) iohexol.

File name: Supplementary Movie 2
Description: Clearing of the MR23 biofilm using 35.2% (w/w) iohexol.

File name: Supplementary Movie 3
Description: Live-cell iCBiofilm imaging of the SE21 biofilm stained with ThT.

File name: Supplementary Movie 4
Description: Live-cell iCBiofilm imaging of the non-stained SE21 biofilm by confocal reflection microscopy.

File name: Supplementary Movie 5
Description: Live-cell iCBiofilm imaging of the SE21 biofilm stained with Mitotracker Deep Red.

File name: Supplementary Movie 6
Description: Live-cell iCBiofilm imaging of the MR23 biofilm stained with Mitotracker Deep Red.

File name: Supplementary Movie 7
Description: Live-cell iCBiofilm imaging of the MR4 biofilm stained with Mitotracker Deep Red.

File name: Supplementary Movie 8
Description: iCBiofilm imaging of the MR23 biofilm treated with PBS (LIVE/DEAD staining). Green and red represent live and dead cells, respectively.

File name: Supplementary Movie 9
Description: iCBiofilm imaging of the MR23 biofilm treated with VCM (LIVE/DEAD staining). Green and red represent live and dead cells, respectively.

File name: Supplementary Movie 10
Description: iCBiofilm imaging of the MR23 biofilm treated with nisin A (LIVE/DEAD staining). Green

and red represent live and dead cells, respectively.

File name: Supplementary Movie 11

Description: Localization of Eap in the MR23 biofilm imaged by iCBilofilm. Magenta and green represent Eap and cells, respectively.

File name: Supplementary Movie 12

Description: Localization of SasG in the MR23 biofilm imaged by iCBilofilm. Magenta and green represent SasG and cells, respectively.

File name: Supplementary Movie 13

Description: Live-cell iCBiofilm imaging of the *C. albicans* biofilm stained with Mitotracker Deep Red.
