# Supplementary material for: Instantaneous Clearing of Biofilm (iCBiofilm): an optical approach to revisit bacterial and fungal biofilm imaging
Source: Commun Biol. 2023 Jan 23;6:38. doi: 10.1038/s42003-022-04396-4 (PMC9870912; doi:10.1038/s42003-022-04396-4)
Supplement: Supplementary file 17 — Reporting Summary [file 42003_2022_4396_MOESM17_ESM.pdf]

nature research

Corresponding author(s): Shinya Sugimoto

Last updated by author(s): Dec 9, 2022

Reporting Summary

Nature Research wishes to improve the reproducibility of the work that we publish. This form provides structure for consistency and transparency in reporting. For further information on Nature Research policies, see our Editorial Policies and the Editorial Policy Checklist.

Statistics

For all statistical analyses, confirm that the following items are present in the figure legend, table legend, main text, or Methods section.

n/a	Confirmed	
☐	☒	The exact sample size (*n*) for each experimental group/condition, given as a discrete number and unit of measurement
☐	☒	A statement on whether measurements were taken from distinct samples or whether the same sample was measured repeatedly
☐	☒	The statistical test(s) used AND whether they are one- or two-sided<br>*Only common tests should be described solely by name; describe more complex techniques in the Methods section.*
☒	☐	A description of all covariates tested
☐	☒	A description of any assumptions or corrections, such as tests of normality and adjustment for multiple comparisons
☐	☒	A full description of the statistical parameters including central tendency (e.g. means) or other basic estimates (e.g. regression coefficient) AND variation (e.g. standard deviation) or associated estimates of uncertainty (e.g. confidence intervals)
☐	☒	For null hypothesis testing, the test statistic (e.g. *F*, *t*, *r*) with confidence intervals, effect sizes, degrees of freedom and *P* value noted<br>*Give P values as exact values whenever suitable.*
☒	☐	For Bayesian analysis, information on the choice of priors and Markov chain Monte Carlo settings
☒	☐	For hierarchical and complex designs, identification of the appropriate level for tests and full reporting of outcomes
☒	☐	Estimates of effect sizes (e.g. Cohen's *d*, Pearson's *r*), indicating how they were calculated

Our web collection on statistics for biologists contains articles on many of the points above.

Software and code

Policy information about availability of computer code

Data collection	NA
Data analysis	Graphpad Prism ver. 9<br>ZEN 3.0 SR Black (64 bit) (Carl Zeiss)<br>Imaris (Bitplane)<br>THUNDER Imaging System (Leica Microsystems)

For manuscripts utilizing custom algorithms or software that are central to the research but not yet described in published literature, software must be made available to editors and reviewers. We strongly encourage code deposition in a community repository (e.g. GitHub). See the Nature Research guidelines for submitting code & software for further information.

Data

Policy information about availability of data

All manuscripts must include a data availability statement. This statement should provide the following information, where applicable:

- Accession codes, unique identifiers, or web links for publicly available datasets
- A list of figures that have associated raw data
- A description of any restrictions on data availability

The authors declare that all data supporting the findings of this study are available within the article and Supplementary Information or are available from the corresponding author upon request.

Field-specific reporting

Please select the one below that is the best fit for your research. If you are not sure, read the appropriate sections before making your selection.

☒ Life sciences ☐ Behavioural & social sciences ☐ Ecological, evolutionary & environmental sciences

For a reference copy of the document with all sections, see nature.com/documents/nr-reporting-summary-flat.pdf

Life sciences study design

All studies must disclose on these points even when the disclosure is negative.

Sample size	Each set of data was from at least three independent experiments
Data exclusions	NA
Replication	Data from at least three independent replicates have been obtained, as indicated in the section of Materials and Methods.
Randomization	NA
Blinding	NA

Reporting for specific materials, systems and methods

We require information from authors about some types of materials, experimental systems and methods used in many studies. Here, indicate whether each material, system or method listed is relevant to your study. If you are not sure if a list item applies to your research, read the appropriate section before selecting a response.

Materials & experimental systems

n/a	Involved in the study	
☐	☒	Antibodies
☒	☐	Eukaryotic cell lines
☒	☐	Palaeontology and archaeology
☐	☒	Animals and other organisms
☒	☐	Human research participants
☒	☐	Clinical data
☒	☐	Dual use research of concern

Methods

n/a	Involved in the study	
☒	☐	ChIP-seq
☒	☐	Flow cytometry
☒	☐	MRI-based neuroimaging

Antibodies

Antibodies used

For immuno-fluorescent microscopy:
Rabbit anti-Eap polyclonal antibody was developed against purified recombinant Eap by Scrum (Tokyo, Japan);
Rabbit anti-SasG polyclonal antibody was developed against purified recombinant SasG by Eurofins Genomics (Tokyo, Japan);
Mouse anti-SasG polyclonal antibodies were developed against purified recombinant SasG by Eurofins Genomics;
Rabbit anti-Curli antibody was developed against purified curli by Scrum;
Mouse anti-dsDNA monoclonal antibody (Abcam, cat#: ab27156);
Alexa 405-conjugated anti-mouse IgG (Thermo Fisher Scientific, cat#: A31553);
Alexa 488-conjugated goat anti-rabbit IgG (Thermo Fisher Scientific, cat#: A32731);
Alexa 647-conjugated goat anti-rabbit IgG (Thermo Fisher Scientific, cat#: A21245);
Alexa 647-conjugated goat anti-mouse IgG (Thermo Fisher Scientific, cat#: A21235);
Alexa Fluor 647 conjugated anti-mouse lymphocyte antigen 6 complex locus G antibody (Ly-6G-Alexa 647) (BioLegend, cat#: 127610)

Validation

Anti-Eap, anti-SasG, and anti-Curli antibodies were validated by our laboratory (Sugimoto et al. J. Bacteriol. 2013; Sugimoto et al. Sci. Rep. 2016; Yonemoto et al. Infect. Immun. 2019). Mouse anti-dsDNA monoclonal antibody, Ly-6G-Alexa 647, and All secondary antibodies were validated by the commercial providers.

Animals and other organisms

Policy information about studies involving animals; ARRIVE guidelines recommended for reporting animal research

Laboratory animals	To isolate neutrophils, C57BL/6 J males and females mice between 8 to 12 weeks of age (CLEA Japan, Inc., Tokyo, Japan) were used. Animals were maintained under specific-pathogen-free conditions at the Jikei University School of Medicine, Japan.
Wild animals	NA

Field-collected samples	NA
Ethics oversight	All animal experiments were approved by the ethics committee of the Jikei University School of Medicine, Japan, and were performed in accordance with approved guidelines and regulations.

Note that full information on the approval of the study protocol must also be provided in the manuscript.
